# Hexagonal Boron Nitride on Liquid and Single‐Crystal Copper: Operando X‐Ray and Atomistic Insights into Growth and Interfacial Structure

**DOI:** 10.1002/advs.76354

**Published:** 2026-07-20

**Authors:** Nikoo Ghanadan, Valentina Rein, Hao Gao, Egor Bersenev, Thomas Sarrazin, Manmeet Kaur Sodhi, Bárbara Canto, Irina Snigireva, Nicolas Gauthier, Anastasios C. Manikas, Costas Galiotis, Maciej Jankowski, Gilles Renaud, Athanasios Dimoulas, Karsten Reuter, Oleg Konovalov, Hendrik H. Heenen, Irene M.N. Groot

**Affiliations:** ^1^ The European Synchrotron Radiation Facility (ESRF) Grenoble France; ^2^ Leiden Institute of Chemistry Leiden University Leiden The Netherlands; ^3^ Fritz‐Haber‐Institut der Max‐Planck‐Gesellschaft Berlin Germany; ^4^ School of Chemistry University of Bristol Bristol UK; ^5^ Université Grenoble Alpes Grenoble France; ^6^ CEA, IRIG/MEM/NRS Grenoble France; ^7^ AMO GmbH Advanced Microelectronic Center Aachen Aachen Germany; ^8^ CEA, LETI Grenoble France; ^9^ Institute of Chemical Engineering Sciences Foundation of Research and Technology Hellas Patras Greece; ^10^ Department of Chemical Engineering University of Patras Patras Greece; ^11^ National Centre for Scientific Research Demokritos Agia Paraskevi Greece

**Keywords:** chemical vapor deposition, copper, density functional theory, dielectric, hexagonal boron nitride, graphene, materials science, molecular dynamics, synchrotron, van der waals force

## Abstract

Two‐dimensional (2D) hexagonal boron nitride (hBN) is a key dielectric for van der Waals nanoelectronics, however, its controlled synthesis by chemical vapor deposition remains challenging and poorly understood. In this context, the growth of hBN on liquid metal catalysts is promising, as the atomically flat liquid surfaces are assumed to promote high‐quality 2D growth, an expectation largely informed by graphene. Here, we implement an involved operando methodology to monitor and quantify hBN growth on molten copper (Liq‐Cu) and re‐solidified single‐crystal copper (SC‐Cu) under near‐ambient‐pressure conditions, enabling real‐time identification of growth stages, morphology, and interfacial structure. Contrary to expectation, Liq‐Cu promotes multilayer and three‐dimensional domain formation, whereas SC‐Cu predominantly yields monolayer‐limited growth. This substrate‐phase dependence correlates with a larger adsorption height of hBN on Liq‐Cu than on SC‐Cu, as determined by X‐ray reflectivity and supported by machine‐learning‐accelerated molecular dynamics simulations. Direct comparison with graphene on Cu further reveals a distinct directional bonding character at the hBN/Cu interface, which rationalizes the observed adsorption‐height trends. More generally, these trends across 2D materials and substrates identify the resulting interfacial stabilization, together with macroscopic factors such as precursor solubility, as a complementary design parameter governing mono‐ vs. multilayer growth in 2D material synthesis.

## Graphical abstract

Liquid metal catalysts are widely assumed to promote high‐quality 2D growth. In this work, operando experiments and machine‐learning‐accelerated simulations show that hBN on Cu breaks this graphene‐derived expectation, where molten Cu promotes multilayer/3D growth, but re‐solidified single‐crystal Cu stabilizes monolayer formation. Interfacial stabilization emerges as a key descriptor, complementary to precursor solubility, for governing layer formation.

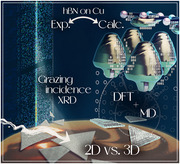

## 1 Introduction

**FIGURE 1:**
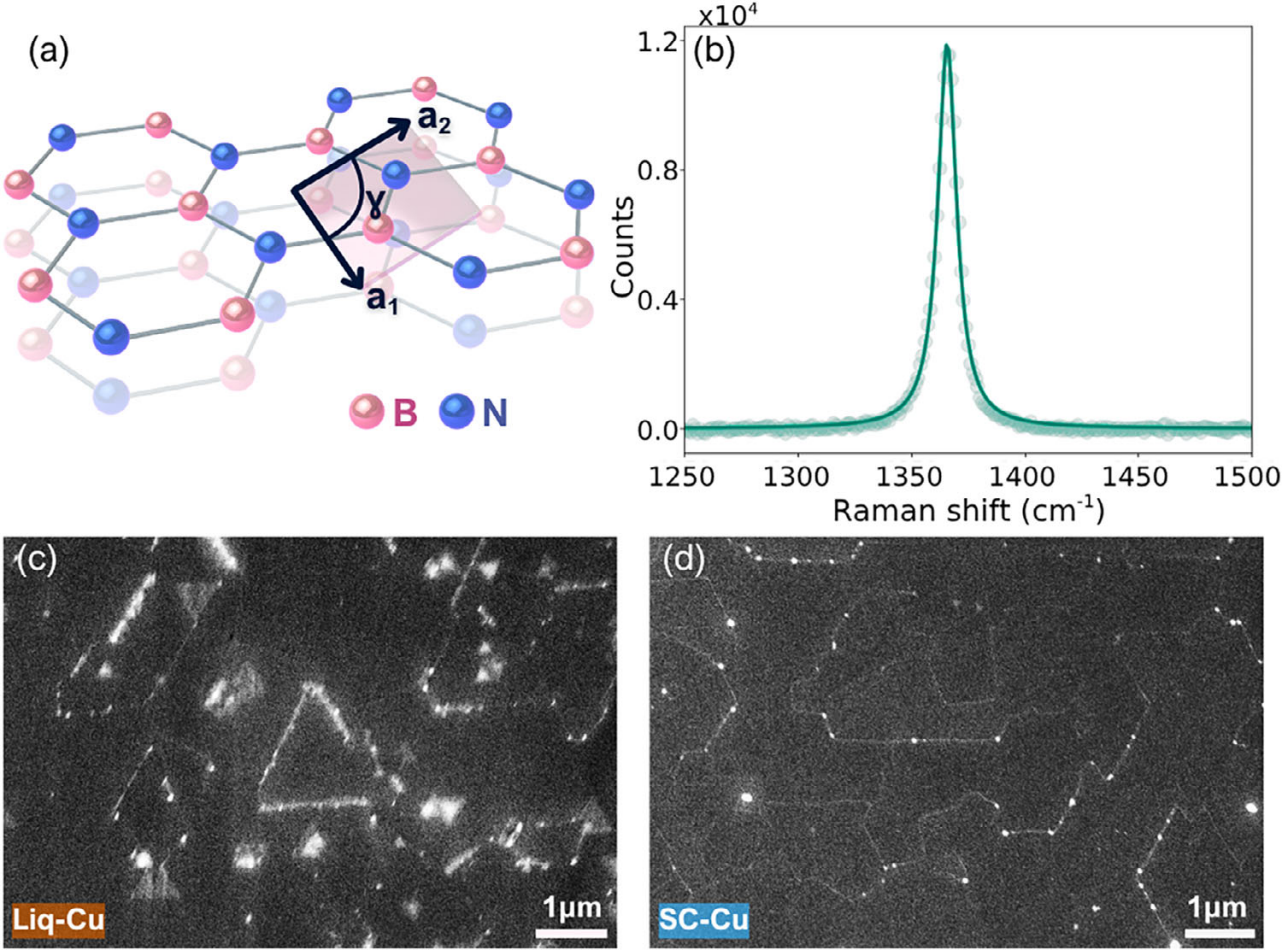
(a) Illustration of the crystallographic structure of hBN atomic layers. (b) Representative Raman spectrum of a transferred hBN sample. The solid line corresponds to the best fit to the Lorentzian peak model centered at $1366\;{\mathrm{cm}}^{-1}$, with a FWHM of $10\;{\mathrm{cm}}^{-1}$. (c,d) Representative ex situ SEM images of as‐grown hBN on Liq‐Cu (c) and SC‐Cu (d).

The isolation of two‐dimensional materials (2DMs) has fundamentally redefined the scope of materials science, unlocking a platform in which electronic, dielectric, and optical functionality can be engineered one layer at a time [1]. Among them, 2D hexagonal boron nitride (hBN) serves as a cornerstone dielectric for van der Waals nanoelectronics [2]. Isostructural to graphene, hBN consists of alternating boron (B) and nitrogen (N) atoms in a honeycomb lattice (Figure 1a). Its wide bandgap, chemical inertness, and thermal stability make it uniquely suited as an ultrathin gate dielectric, tunneling barrier, and encapsulation layer in 2D heterostructures [3, 4, 5]. In addition, hBN supports hyperbolic phonon‐polaritons for nanophotonics [6] and can withstand aggressive processing environments, expanding its relevance from electronics to energy applications and protective coatings [7, 8]. Despite its broad potential, the widespread application of hBN is limited by the lack of scalable synthesis routes of high‐quality, homogeneous films, a challenge that ultimately stems from an incomplete mechanistic understanding of hBN's growth kinetics.

Bottom‐up chemical vapor deposition (CVD) is widely regarded as the most promising scalable synthesis route to large‐area mono‐/few‐layer hBN as it can, in principle, tune thickness, orientation, and morphology through catalyst choice, precursor chemistry, and growth parameters (temperature, pressure, flow, and feedstock activation) [8, 9]. Most commonly, hBN CVD is performed on transition‐metal catalysts, predominantly Cu‐ and Ni‐based substrates, at low‐pressure to atmospheric‐pressure growth windows. Recent breakthroughs toward large‐area single crystallinity include epitaxial growth of a 10×10 ${\mathrm{cm}}^{2}$ single‐crystal hBN monolayer on Cu(110) vicinal foils, and single‐crystal hBN monolayers on Cu(111) thin films across two‐inch sapphire wafers [10, 11].

Despite rapid progress, scalable CVD of hBN remains limited in reproducibility compared with what has been achieved for graphene. Wafer‐scale single‐crystal graphene has been realized via both single‐nucleus growth and stitching routes, whereas hBN growth has more often relied on unidirectional alignment and stitching strategies, with domain sizes remaining in the micron‐scale, highlighting additional obstacles specific to hBN [10, 12]. Reviews emphasize that hBN growth is intrinsically more constrained than elemental 2DMs, because it is a compound crystal. Accordingly, maintaining B:N stoichiometry and controlling stacking order during multilayer growth are central challenges that directly impact dielectric breakdown, defect density, and device‐grade uniformity [9]. Furthermore, the lower symmetry of hBN promotes antiparallel domains and twin boundaries on many common substrates [10]. Growth mechanisms are also strongly substrate‐dependent. On Cu, hBN growth appears more complex than a purely surface‐mediated adsorption‐dehydrogenation pathway, with the literature pointing to strong B/N chemical‐potential effects at island edges and possible subsurface/bulk participation during growth, which complicate layer‐number control [13]. On Ni, by contrast, growth can be governed by precipitation, while lattice mismatch on other substrates can introduce non‐classical aggregation behavior [13]. This complexity is compounded by the mosaicity, grain boundaries, and anisotropy of frequently employed polycrystalline catalysts, which exacerbate uncontrolled nucleation and substrate‐induced defects in the hBN adlayer, and which also undermine reproducibility across different setups and laboratories [14]. As a result, nominally similar recipes can yield different nucleation densities, growth rates, and thickness distributions. Many mechanistic hypotheses are based on post‐growth ex situ characterization, in which cooling leads to thermal contraction and substrate restructuring, introducing strain, ridges, delamination features, and morphological changes that obscure the true growth‐stage interface and kinetics [9, 13, 15]. Accordingly, claims of wafer‐scale monolayer uniformity require spatially resolved mapping and thickness‐sensitive validation, rather than reliance on a single ex situ indicator or local probe measurement alone.

Growth of hBN on single‐crystal metal substrates reduces defects inherent to polycrystalline foils and can suppress uncontrolled nucleation and orientation spread [16, 17]. However, large‐area single‐crystal substrates remain costly and time‐consuming to prepare, motivating the development of alternative catalyst platforms [11]. In this context, liquid metal catalysts (hereby denoted LMCats), such as molten Cu, Ga, Au, and alloys, have emerged as attractive growth substrates for 2DMs due to their surfaces being atomically smooth, isotropic, and dynamically self‐healing, thereby eliminating fixed steps and grain boundaries [18]. In the most‐studied case of graphene, liquid Cu has enabled reduced nucleation density and rapid lateral growth, producing large (mm‐sized) single‐crystal domains [19, 20]. Similarly, hBN growth on LMCats has also been demonstrated, including wafer‐scale single‐crystalline monolayer hBN on liquid Au and orientation‐controlled growth on liquid Cu [21, 22]. However, whether LMCats are universally beneficial for hBN, as they are for graphene, is not yet established.

A major obstacle to resolving these questions is the scarcity of real‐time operando measurements that directly probe growth kinetics and interface formation under realistic (ambient‐ and low‐pressure) CVD conditions. The limited number of studies that directly track hBN CVD in situ (e.g., via in situ X‐ray photoelectron spectroscopy (XPS) or X‐ray diffraction (XRD)), do so under vacuum, which leaves the growth kinetics and interface evolution constrained to impractical catalyst phases and growth windows [23]. Without operando data, it is difficult to isolate competing explanations for (i) why hBN growth tends toward monolayer self‐termination on some catalysts (e.g., Cu‐ or Pt‐based growth) [11, 24] yet becomes multilayer/3D on others where segregation/precipitation pathways may dominate (e.g., Fe‐, Ni‐, or Ni‐Fe‐based catalysts) [25, 26], (ii) how the catalyst phase (liquid vs solid) modifies the adsorption geometry and effective bonding character, and (iii) how these microscopic factors translate into distinct macroscopic growth regimes. Addressing this gap requires correlating operando growth signatures with quantitative interfacial descriptors that can be compared to atomistic simulations.

Here, we extend an operando methodology previously established for graphene [19, 20, 27] and apply it to hBN growth on both molten and re‐solidified single‐crystal Cu (hereby denoted Liq‐Cu and SC‐Cu, respectively). In situ, we track the growth process using radiation‐mode optical microscopy (Rad‐OM) and resolve film morphology and structure via synchrotron grazing‐incidence X‐ray diffraction (GID) and X‐ray reflectivity (XRR) under near‐ambient‐pressure growth conditions. GID probes characteristic two‐dimensional Bragg rods (BRs) [28, 29], providing access to crystallinity, in‐plane orientation, and domain size, while XRR yields electron‐density profiles normal to the interface [30] and enables quantitative determination of layer thickness and the hBN/Cu interfacial structure [27]. Complementary post‐growth XPS, Raman spectroscopy, atomic force microscopy (AFM), and scanning electron microscopy (SEM) verify hBN composition and film integrity ex situ. By combining operando XRR with atomistic simulations based on machine‐learned interatomic potentials trained on first‐principles data, we directly link experimentally resolved interfacial geometries, which are characterized by a van der Waals separation gap [31, 32, 33, 34], to microscopic 2DM/substrate interactions. This multimodal approach reveals a pronounced substrate‐phase dependence of hBN growth, where Liq‐Cu unexpectedly promotes multilayer and three‐dimensional domain formation, while SC‐Cu predominantly supports higher‐quality, monolayer‐terminating growth with larger crystalline domains. A comparison with graphene suggests that this behavior correlates with interfacial layer adhesion, as captured by the separation gap, instead of solely the substrate phase. The interfacial stabilization emerges thus as a microscopic factor that, together with macroscopic considerations such as precursor solubility in Cu, may influence mono‐ vs. multilayer growth.

## 2 Methods

### 2.1 Sample Preparation and CVD Growth

The CVD substrate samples consisted of Cu foils that were placed inside the reactor [35] on a chemically etched tungsten support, which promotes wetting and stabilizes the molten Cu droplet (see Figure S1 for more details). The Cu foils were melted and annealed at 1400 K under a constant flow of $H_{2}$ at 30 sccm for ∼30 min. During growth, reactor conditions were maintained at 200 mbar pressure, ∼1370 K sample temperature, and a carrier gas flow of 200 sccm Ar and 20 sccm $H_{2}$. Ammonia borane (AB) powder was used as the precursor [8]. The AB was sublimed at 60
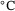
–120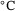 from a cell equipped with a proportional‐integral‐derivative (PID)‐controlled heating element and delivered using an auxiliary Ar flow (up to 50 sccm) through a dedicated line, or alternatively through the main line at higher flow rates (220 sccm). Throughout this work, “AB exposure time” *t* is measured relative to the onset of precursor delivery. Specifically, *t* = 0 is defined as the moment when the AB cell temperature first exceeds 60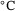, and all subsequent times are measured from this reference point. For the growth on the SC‐Cu substrate, the molten samples were cooled down slowly (∼1 degree per minute), solidified, and a temperature below Cu's melting temperature (∼1355 K) was maintained during growth. A dominant Cu(111) surface orientation of the SC‐Cu substrates was verified by GID.

### 2.2 Real‐time Growth Control

The hBN growth process was monitored in real time through the CVD reactor's [35] optical window using Rad‐OM [36], a technique that provides image contrast based on the emissivity difference between the substrate and the 2DM overlayer. Images were taken with a *Basle* *r* CMOS camera. We note that Rad‐OM images from Liq‐Cu were background‐subtracted, averaged over three frames, and Gaussian‐smoothed to improve contrast and reduce noise. Unedited data are shown in the Supporting Information. Once the AB cell temperature exceeded 60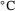, it was ramped up at a rate of 1–2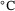/min until a rapid growth surge (1–5 s) was observed (see the Supporting Information for the run‐by‐run precursor exposure conditions). We note that quenching the precursor upon layer formation typically induces etching of said layer, resulting in its complete removal within 5–10 min.

### 2.3 Ex Situ Characterization

For ex situ measurements, upon layer detection by Rad‐OM, the precursor supply was reduced and the substrate rapidly cooled (or, in the case of liquid substrates, allowed to solidify first before cooling) before etching occurs to preserve the layer state for analysis. XPS measurements on as‐grown hBN were carried out using the *PHI VersaProbe II* Scanning XPS Microprobe with a 1486.6 eV monochromatic X‐ray source. The B 1s and N 1s core‐level XPS spectra were both measured with a photon energy of 1.49 keV, with 0.6 eV spectral resolution on spot sizes of 200, 100, 50, and 20 μm. SEM data shown in Figure 1c,d were recorded using the *Zeiss LEO 1530 Oxford X‐Max* with a beam energy of 15.00 keV. For AFM measurements and Raman spectroscopy analysis, the samples were transferred onto thermally grown 90 nm ${\mathrm{SiO}}_{2}$/Si substrates. For the AFM measurements, the transfer was performed using a wet‐transfer process based on a 3 M NaOH solution [37, 38]. For Raman analysis, the samples were transferred using the standard poly(methyl methacrylate) (PMMA)‐assisted wet‐transfer method [39]. AFM measurements were carried out in tapping mode using a *Bruker Corporation Dimension Icon* AFM equipped with an *OTESPA* cantilever tip (force constant: 26 N/m; resonant frequency: 200–400 kHz), in air at room temperature. Raman spectra were collected using a *Renishaw InVia* spectrometer equipped with a 514 nm excitation laser, a power of 0.5 mW, and an acquisition time of 10 s. A 24 × 16 μ $m^{2}$ area was mapped with a step size of 1 μm to evaluate sample quality. Spectral data analysis was performed using Renishaw Wire 4.0 software, with hBN peaks fitted to a single Lorentzian function.

### 2.4 In Situ Characterization

Synchrotron X‐ray measurements were carried out at the ID10‐SURF beamline of the European Synchrotron Radiation Facility (ESRF, France) [40, 41, 42]. The Be window of the CVD reactor allowed X‐ray beam penetration with minimal attenuation. Measurements utilized a monochromatic beam with energies of 22 keV and 22.5 keV with a vertical size of 16 μm and a horizontal size of 25 μm (FWHM). The detector used was a 2D Maxipix detector with CdTe sensor and pixels of 55 μm size with a sample‐to‐detector distance of 690 mm. A double crystal deflector was used to change the incident angle θ on liquid surfaces [43, 44]. The reflectivity data were reconstructed from the experimental images on the 2D detector, taking into account the curvature of the liquid metal surface following the approach outlined by Konovalov et al. [45], and were fitted using the Refl1D software [46]. The density of liquid Cu ($\rho_{\text{Cu}}$) at 1370 K was fixed to 2.21 $e^{-}$Å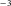 ($\rho =7.95\;g\cdot {\mathrm{cm}}^{-3}$) [47]. The thickness of a single BN layer ($d_{\mathrm{hBN}}$) was fixed to 1.45 Å [8] and its density ($\rho_{\mathrm{hBN}}$) was derived from the density of bulk hBN and the interlayer spacing extracted from the [001] Bragg peak [48]. The interfacial separation distance/absorption height ($d_{\mathrm{gap}}$) was calculated as the sum of the void layer thickness between the Cu and hBN, ($d_{\text{void}}$), and half the thickness of the hBN layer ($d_{\mathrm{hBN}}$) in the three‐slab model. For GID measurements, Bragg rods (BRs) were recorded by positioning the detector at the expected diffraction angle while rotating the reactor around the azimuthal axis, perpendicular to the sample surface, upon formation of the layer(s) [49]. BR images shown in this study represent an accumulation of multiple images capturing scattering from individual grains during azimuthal rotation. The individual scans comprising BRs were projected onto 3D reciprocal‐space maps using the BINoculars binning algorithm [50], where each point in the resulting azimuthal $q_{x}-q_{y}$ map denotes the contribution of a distinct crystallite.

### 2.5 Training of the Machine‐Learned Interatomic Potential

To describe the hBN/Cu system via atomistic simulations, we fitted a moment tensor potential (MTP) [51] with hyperparameters of level = 20 and a 6 Å cutoff radius. The potential was trained to density functional theory (DFT) calculations based on the Perdew–Burke–Ernzerhof (PBE) exchange‐correlation functional [52] and the non‐local many‐body dispersion (MBD‐NL) correction [53] as implemented in FHI‐aims [54]. The electronic structure calculations were converged to $10^{-6}$ eV using a K‐point density exceeding 60/Å or a single K‐point along the vacuum direction for slab models. Lattice parameters and atomic positions were relaxed until residual forces fell below 1 meV/Å  and 10 meV/Å, respectively. These DFT settings are consistent with previous work demonstrating a very accurate description of graphene (Gr) on SC‐Cu and Liq‐Cu [27] and also reproduce the hBN lattice constant within 0.5%, ensuring high‐quality reference data. The hBN/Cu training set was derived from a Gr/Cu dataset of the same previous work [27], and the resulting potential was trained with comparable accuracy. Further details on the training procedure are summarized in the Supporting Information.

### 2.6 Molecular Dynamics Simulations

Molecular dynamics (MD) simulations were performed to characterize the interface of hBN on the Cu substrates employing the trained hBN/Cu MTP. For comparison, we conducted analogous simulations for Gr on the same interfaces using a Gr/Cu MTP from our earlier work [27]. To model the interface realistically, we constructed periodic slab models comprising the 2D materials adsorbed on the respective substrates. As detailed in the Supporting Information, the slab geometries were chosen to minimize residual strain arising from the incommensurate lattice constants of the 2D materials and the substrates. The resulting models contained ∼1700 atoms for Liq‐Cu and ∼5000–15000 atoms for SC‐Cu with the residual strain kept below ±0.4% which we verified to have no impact on the results. The temperature‐dependent lattice parameters for face‐centered cubic (fcc) Cu and free‐standing hBN and Gr were converged in separate NPT simulations, while the slab models were treated in the NVT ensemble. All MD simulations were run for 1 ns with a 1 fs time step, which provides plentiful statistics for the target observables [27]. The simulations are based on a Nose‐Hoover thermostat [55] with an additional Parrinello‐Rahman barostat [56] in the case of NPT simulations, using damping parameters of 0.1 ps. Slab models of Liq‐Cu were simulated at 1370 K and SC‐Cu at 1300 K (and for comparison at 1200 K). The lattice parameters were converged for the same temperatures while maintaining 1 atm pressure.

Analogous to our previous work [27], we process the $d_{\mathrm{gap}}$ (separation distance between hBN and Cu) from our MD trajectories via the approximated electron density profile along the surface normal. The electron density profile is derived from the averaged atom density via Gaussian smearing, where the area of the Gaussian corresponds to the number of electrons (29 for Cu, 4 for B, 7 for N, and 6 for C) and σ values correspond to the experimentally determined roughness factors.

## 3 Results

### 3.1 Composition and Morphology of hBN Layers

We establish the chemical identity, morphology, and integrity of the CVD‐grown films on Liq‐Cu and SC‐Cu using complementary ex situ characterization. Raman spectroscopy and XPS support the assignment of stoichiometric, crystalline‐quality hBN, while SEM and AFM reveal a pronounced substrate‐phase dependence in post‐growth morphology. Thickness, crystalline structure, and interfacial geometry are quantified primarily by the operando XRR/GID analysis.

#### 3.1.1 XPS

Ex situ XPS was used as a supporting probe of chemical identity of the as‐grown layers. The N 1s and B 1s core levels are centered at 190.4 eV and 397.9 eV respectively, in agreement with reported values for stoichiometric hBN [57, 58]. Peak fitting yields an atomic ratio of N:B = 0.98. As expected for an ex situ, surface‐sensitive measurement, additional carbon and oxygen‐containing surface species were also detected (See Figure S2 for details).

#### 3.1.2 Raman Spectroscopy

After transfer to $\mathrm{Si}/{\mathrm{SiO}}_{2}$ wafers, the hBN samples were characterized by Raman spectroscopy. Figure 1b shows a representative Raman spectrum, and Figure S3 presents a Raman map of the $E_{2g}$ phonon mode's peak position and the corresponding full width at half maximum (FWHM). The $E_{2g}$ mode, which corresponds to the in‐plane vibrations of B and N atoms, is the primary Raman‐active mode of hBN and reflects the material's hexagonal symmetry and strong covalent bonding [59, 60, 61, 62]. Here, we found its average peak position around 1366 ${\mathrm{cm}}^{-1}$, which is in good agreement with the literature [59, 60, 61, 62] which, together with a narrow FWHM of the peak (10 ${\mathrm{cm}}^{-1}$) [62, 63], is characteristic of highly pure and crystalline hBN, with minimal compressive/tensile strain or defect contributions. Unlike graphene, hBN's layer thickness cannot be reliably inferred from its Raman shift, as the peak position shows only weak dependence on thickness due to weak interlayer coupling [59, 64].

#### 3.1.3 SEM

SEM on the as‐grown samples reveals a clear substrate‐phase dependence of the hBN morphology, as illustrated in Figure 1c,d. In the SEM images, regions of weaker contrast are consistent with laterally continuous ultrathin hBN coverage, while bright local features typically correspond to raised multilayer/3D outgrowths and/or local surface topography variations (i.e., contrast is not thickness‐calibrated) and bright networks indicate coalescence/defect lines where domains impinge. On Liq‐Cu, we observe a higher density of bright protrusions and triangular/pyramidal outgrowths, often decorating coalescence lines, indicating a stronger tendency toward multilayer/3D growth under otherwise comparable conditions. On SC‐Cu, the surface is instead dominated by laterally merged multifaceted domains with far fewer bright outgrowths, consistent with a predominantly monolayer‐terminating regime. This interpretation is supported by post‐transfer AFM measurements (Figure S4), which show substantially greater topographical relief for samples grown on Liq‐Cu than for those grown on SC‐Cu. Additional representative SEM images highlighting these differences are provided in Figure S5. Similar morphologies and shape evolution, including triangular partial coverage and truncated or multifaceted domain coalescence, have been reported previously for hBN CVD on both solid [17, 65, 66] and liquid [22] Cu, although such variations are generally attributed to growth conditions rather than substrate phase.

### 3.2 In Situ Growth Monitoring

#### 3.2.1 Optical Microscopy

**FIGURE 2:**
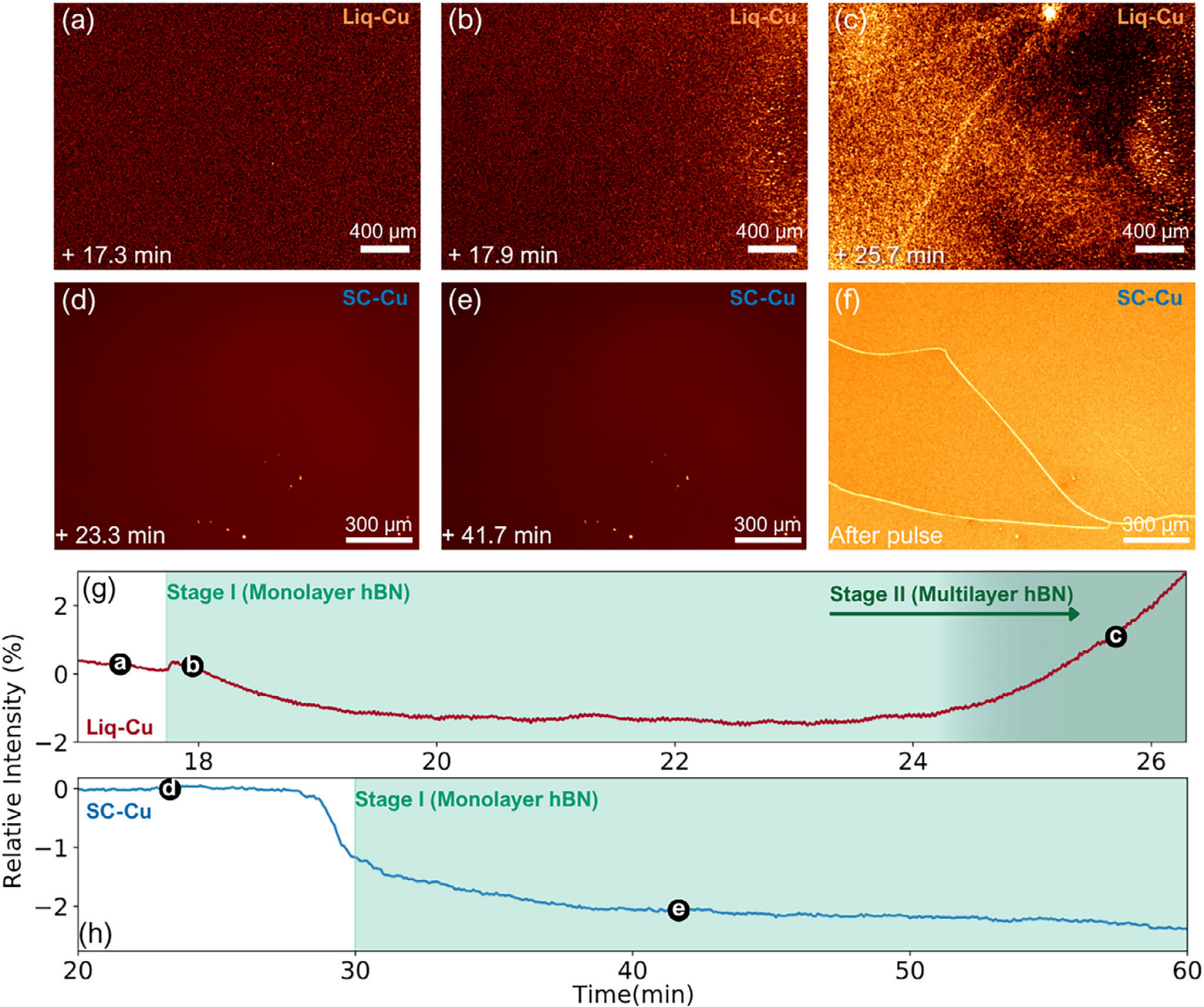
In situ Rad‐OM measurements during growth: (a–c) False‐color, background‐subtracted snapshots of hBN on Liq‐Cu, showing (a) the bare Liq‐Cu surface, (b) a monolayer hBN film with few 3D islands (white spots, right‐hand corner), and (c) multilayer hBN. (d–f) Corresponding false‐color, background‐subtracted snapshots on SC‐Cu before growth (d), after monolayer film growth (e) and multilayer growth induced by a high‐flux precursor pulse (f). (g,h) Background‐normalized average image intensity as a function of time on Liq‐Cu and SC‐Cu, respectively. The signals have been corrected for multiplicative thermal drift. The time point t=0 corresponds to the moment at which the AB precursor cell temperature exceeds 60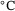. The shaded region (green) marks the time after which monolayer hBN is detected via X‐ray characterization.

We use Rad‐OM in tandem with X‐ray scattering to monitor and control growth in real time. Rad‐OM exploits emissivity differences between the Cu surface and the 2D overlayer at high temperature, and has proven effective for graphene growth tracking, where individual domains are readily resolved and emissivity contrast is prominent [19, 36]. However, for hBN, the optical contrast is weaker, and typical domain sizes are at or below our camera resolution of ∼1 μm (see Figures S6 and S7 as well as Movies S1 and S2). We therefore track growth via the spatially averaged, background‐normalized intensity as shown in Figure 2g,h, which provides a semi‐global signature of coverage changes [19, 67].

On Liq‐Cu, an initial rapid transition in average intensity (marked as “Stage I Monolayer hBN/Cu” in Figure 2g) occurs spontaneously when the AB cell reaches the high sublimation‐temperature regime (above 85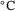), producing a small transient intensity increase (0.1%–0.2%) followed by a net decrease (1%–2%) relative to the bare‐Cu baseline. In situ X‐ray measurements confirm that this stage corresponds predominantly to 2D hBN formation. Continued precursor exposure (“Stage II Multilayer hBN/Cu” in Figure 2g) leads to the emergence of irreversible optically bright features on top of the initial layer (c.f. Figure 2c), consistent with multilayer/3D outgrowth, as corroborated by XRR below. This multilayer growth contrasts graphene CVD on Liq‐Cu, which typically exhibits an effectively monolayer‐limited growth under comparable conditions, as shown in our prior operando studies [19, 20, 27].

On SC‐Cu, a similar Rad‐OM signature is detected, with the average emitted intensity decreasing upon growth of the layer (see Figure 2h). However, no distinct features can be resolved directly in the images. In contrast, multilayer growth on SC‐Cu could be detected in Rad‐OM as the appearance of bright spots with intensities comparable to those observed on Liq‐Cu. In our experiments, this multilayer mode was only triggered by applying a brief high‐flux precursor pulse after establishing an initial monolayer (Figure 2f).

#### 3.2.2 X‐Ray Reflectometry

**FIGURE 3:**
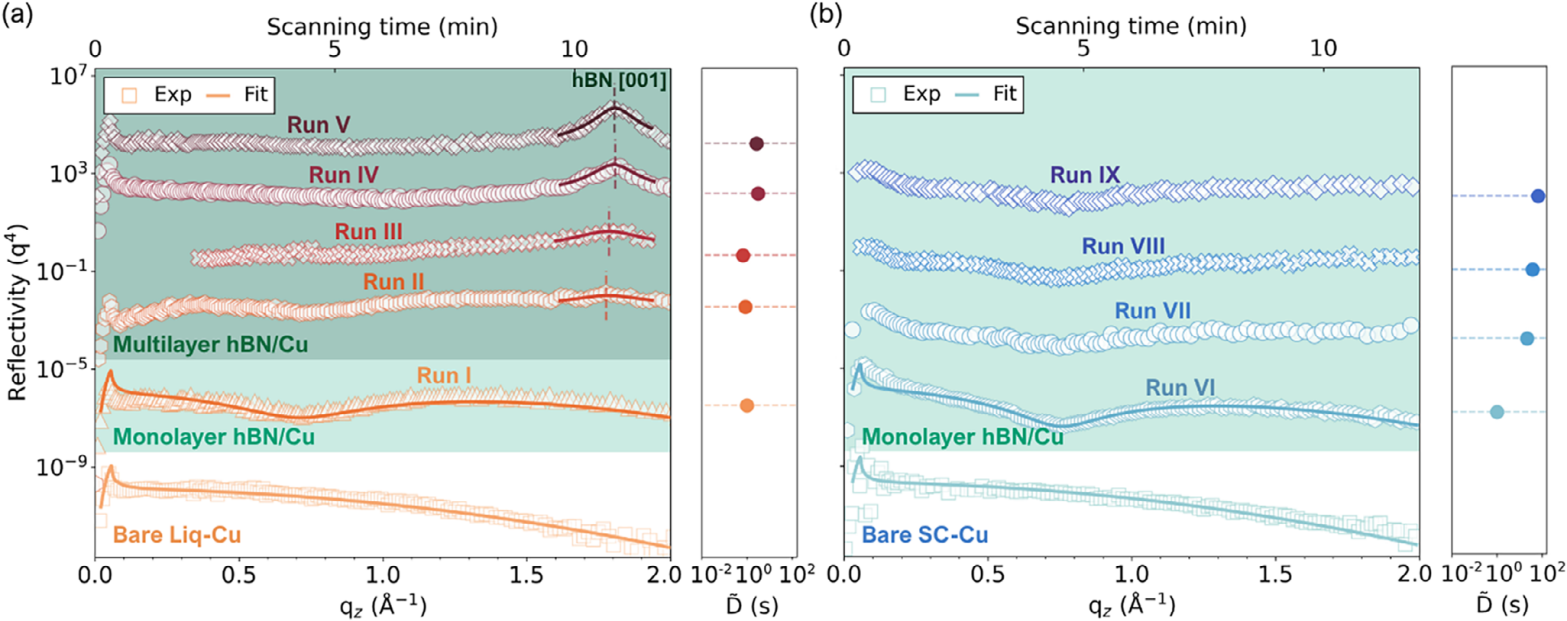
In situ XRR profiles of bare and hBN‐covered Cu samples corresponding to separate runs at various stages of growth (a) on Liq‐Cu and (b) on SC‐Cu. The bottom‐most curves correspond to the bare Cu surfaces prior to growth. The curves above correspond to separate hBN growth runs with different AB exposure histories. Solid lines correspond to best fits. The top axis indicates time in minutes, corresponding to the average scan acquisition duration. For clarity, the data are shown in the conventional $q_{z}^{4}$‐weighted representation, i.e., *R*($q_{z}$)*$q_{z}^{4}$ and are offset vertically. The right‐hand side markers indicate corresponding AB exposure metric $\tilde{D}$ for each scan; details of its calculation are provided in the Supporting Information.

Our XRR measurements enable in situ detection of ultrathin interfacial hBN layers and can distinguish mono‐ from multilayer formation through characteristic diffraction features which are revealed by a probe that is representative of the surface film as a whole, owing to its inherent lateral and temporal averaging. The grazing‐incidence footprint spans hundreds of micrometers across the curved Liq‐Cu surface (as opposed to tens of microns in the non‐grazing regimes), providing mesoscale lateral averages of coverage and roughness [45, 68]. In addition, the acquisition time of a single scan (∼12 min; top horizontal axis in Figure 3) exceeds the timescale of initial layer formation on Liq‐Cu and rather matches that of multilayer and three‐dimensional feature development, such that XRR primarily resolves the average growth stages.

Figure 3 summarizes XRR profiles for Liq‐Cu and SC‐Cu recorded in independent growth runs under different AB source temperature/time profiles. The corresponding onset‐normalized precursor exposure times are indicated next to each curve (see Figures S8 and S9 for more details). On both substrates, low precursor doses lead to the emergence of a pronounced minimum near $q_{z}\approx 0.75\;{Å}^{-1}$, which indicates monolayer‐level hBN coverage (Stage I), as confirmed by our modeling (c.f. Subsection 3.4) and in agreement with analogous signatures reported for graphene on Cu ($q_{z}\approx 0.8\;{Å}^{-1}$) [19, 27, 69]. The sparse (<20%) formation of pyramidal outgrowths on this monolayer film cannot be excluded, as such geometries barely contribute to specular reflectivity when their areal coverage is low (compare Figure S10 for more details).

Multilayer growth is observed exclusively on Liq‐Cu, where higher precursor doses lead to a suppression of the monolayer minimum and the appearance of an out‐of‐plane Bragg peak near $1.80\;{Å}^{-1}$, which is characteristic for coherent multilayer formation [70]. For intermediate precursor doses (Run II, Figure 3), this Bragg feature can coexist with the minimum feature which indicates that multilayer and monolayer contributions are captured in a single scan. The Bragg feature is assigned to a [001] stacking, and yields interlayer spacings of 3.52–3.48 Å (Stage II, see Table S1 and Figure S11), which are larger than the ∼3.33 Å room‐temperature bulk value [8], and consistent with hBN's positive out‐of‐plane thermal expansion at the growth temperature [71]. At high precursor doses, we observe an increasing multilayer thickness (≈6–16 layers), which is accompanied by a modest reduction of the interlayer spacing, as evidenced by the [001] peak shift. This reduction is in line with prior reports attributing thickness‐dependent interlayer spacing variations to nanoconfinement [72, 73], which, in this case of a liquid surface, is an intrinsic stacking effect unrelated to substrate‐imposed strain [19].

### 3.3 Crystalline Structure and In‐Plane Ordering

**FIGURE 4:**
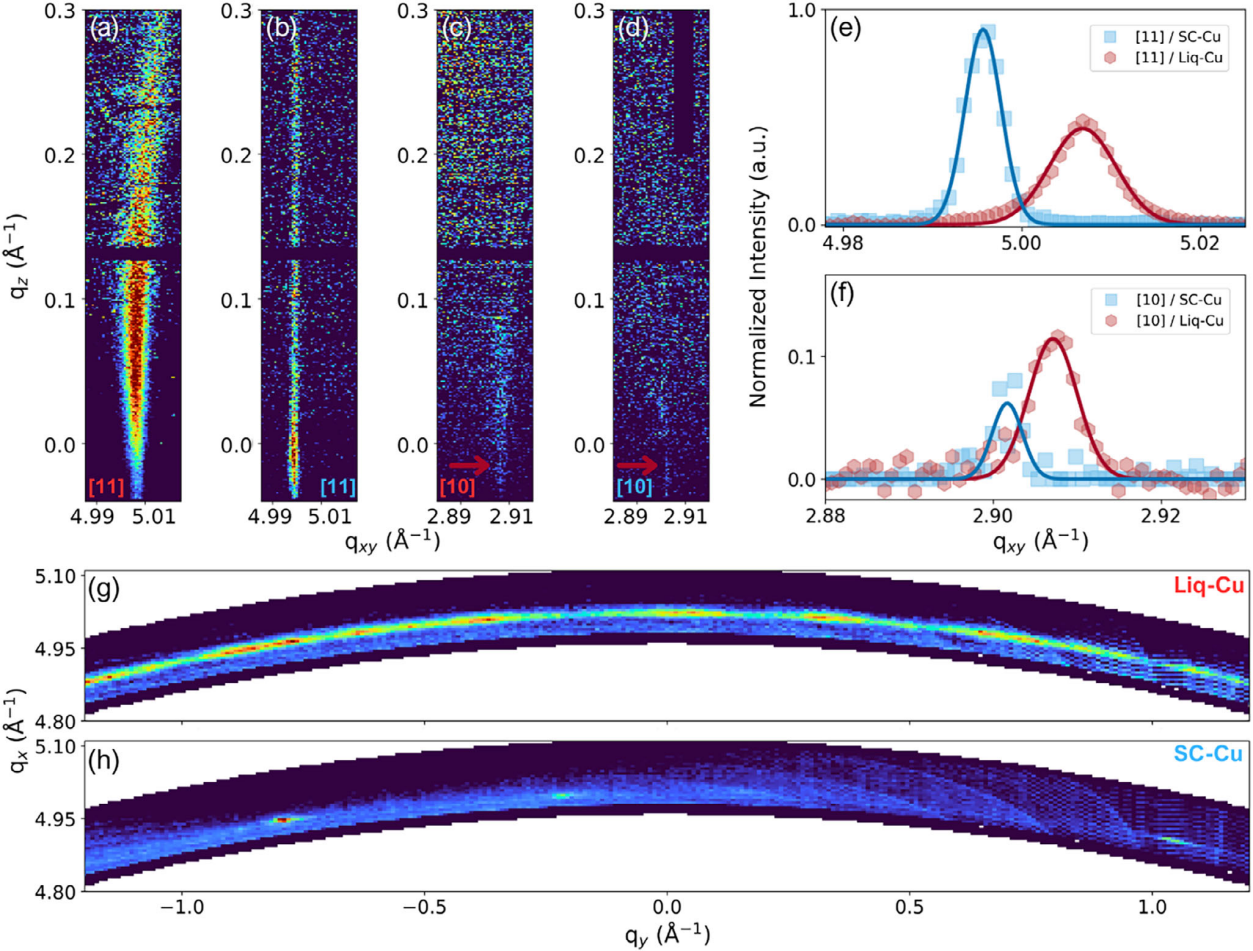
Reciprocal space detector images of hBN [10] and [11] 2D BRs for layers grown on Liq‐Cu (a: [11], c: [10]) and SC‐Cu (b: [11], d: [10]). Each image is a composite assembled from multiple measurements taken while rotating the sample. BRs in c and d are indicated with a red arrow. (e,f): Integrated 1D profiles for [11] and [10] BRs, respectively, with Gaussian fits shown as solid lines. (g, h): In‐plane (azimuthal) projections of the [11] BRs shown in (a,b), respectively. Intensities are summed over $q_{z}$, yielding a $q_{x}-q_{y}$ heat map where spots correspond to individual diffracting grains and their in‐plane positions. Masked area in (c) corresponds to SC‐Cu crystal truncation rods.

We use in situ GID to obtain a uniquely direct structural fingerprint, allowing us to quantify in‐plane lattice parameters, domain size, and orientational ordering of the growing hBN and clearly distinguish the Cu substrates. On the bare substrates, grazing‐incidence scattering confirms the expected signatures: a broad liquid‐like diffuse peak on Liq‐Cu and sharp Bragg reflections from the re‐solidified SC‐Cu (See Figures S12–S14). After growth, the presence of an ultrathin 2D hBN layer is identified by continuous 2D Bragg rods (BRs), analogous to graphene [19]. Figure 4a–d shows the reciprocal‐space maps [50] acquired at the expected in‐plane positions of the hBN [11] and [10] BRs while rotating the sample azimuthally (thus yielding an average BR from multiple hBN grains), where $q_{\mathrm{xy}}$ and $q_{z}$ denote in‐plane and out‐of‐plane momentum transfers, respectively. On SC‐Cu, the [11] BR is narrow and well defined (as shown in Figure 4b and Movie S4), whereas on Liq‐Cu it appears as a slightly curved (“bowed”) trace composed of multiple short segments (see Figure 4a and Movie S3). Physically, this means that a layer of hBN grown on SC‐Cu has the signature of a thin, highly ordered layer, whereas a layer of hBN grown on Liq‐Cu consists of a large number of smaller, less ordered crystals with slightly differing orientations with respect to one another. Presence of vertical [10] BRs (Figure 4c for Liq‐Cu and Figure 4d for SC‐Cu) confirms the hexagonal ordering of the grown hBN, exhibiting width and $q_{\mathrm{xy}}$‐position consistent with the observation of [11] BRs. These BRs are indicated with a red arrow to improve visibility. Note the very low intensity of these BRs, which is ca. 10% higher than the background scattering, as shown in Figure 4f. This substrate‐related difference is consistent with the azimuthal projections of the [11] rod: Liq‐Cu (Figure 4g) yields a near‐continuous arc, characteristic of a highly polycrystalline (powder‐like) in‐plane orientation distribution, while SC‐Cu (Figure 4h) shows only a small number of discrete orientations, consistent with a more ordered (epitaxially aligned) film with larger crystalline domains. Full 3D reciprocal‐space projections are shown in Figures S15 and S16.

Signals from the Refs. [11] and [10] maps were integrated to obtain 1D profiles and fitted with Gaussian functions (Figure 4e,f), with the corresponding parameters listed in Table 1). On average, BRs of the [11] family appear at $q_{\mathrm{xy}}=4.994\pm 0.027\;{Å}^{-1}$ (Liq‐Cu) and $q_{\mathrm{xy}}=4.998\pm 0.027\;{Å}^{-1}$ (SC‐Cu), while the weaker [10] BRs appear at $q_{\mathrm{xy}}=2.892\pm 0.017\;{Å}^{-1}$ (Liq‐Cu) and $q_{\mathrm{xy}}=2.902\pm 0.017\;{Å}^{-1}$ (SC‐Cu) (see details in the Figures S17 and S18 and Tables S2 and S3). These BR values correspond to average in‐plane hBN lattice parameters of $a_{1}=2.514\pm 0.006\;Å$ (Liq‐Cu) and $a_{1}=2.510\pm 0.008\;Å$ (SC‐Cu). The extracted $a_{1}$ values are within the range reported for hBN under ambient conditions [74] (Figure S19) and differ only weakly between SC‐Cu and Liq‐Cu, suggesting that the layers are not strongly strained on average. Similar to graphene, hBN exhibits a weak, negative in‐plane thermal expansion over a broad temperature range, thus increases of $a_{1}$ at the growth temperature are not expected [19, 71].

**TABLE 1:** Averaged experimental values for the scattering vector $q_{\mathrm{xy}}$, lattice parameter $a_{1}$, and crystalline domain size for [11] and [10] BRs. Domain sizes are estimated from BR FWHM (Scherrer approximation) and are limited by instrumental reciprocal‐space resolution; values reflect the broadened/resolvable domain population. See Supporting Information for discussion of uncertainties.

Substrate	Liq‐Cu		SC‐Cu	
BR indices	[11]	[10]	[11]	[10]
$q_{xy}$ [Å 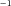 ]	4.994 ± 0.027	2.892± 0.017	4.998± 0.027	2.902 ± 0.017
$a_{1}$ [Å]	2.514 ± 0.006		2.510 ± 0.008	
FWHM [Å 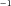 ]	0.007 ± 0.001		0.003 ± 0.001	
Domain size [μm]	0.090 ± 0.029		0.203 ± 0.097	

The BR widths (FWHM) provide an estimate of the lateral crystalline domain size via the Scherrer approximation [75]. It should be noted that in our experiments the q‐space resolution was limited by the pixel size of the detector. Thus, rods from sufficiently large crystallites may be resolution‐limited, and their intrinsic widths cannot be extracted. The reported Scherrer sizes represent an effective coherence length of the broadened (resolvable) part of the distribution and should be regarded as a lower‐bound estimate for the domain size, without corresponding directly to the lateral feature sizes observed in SEM, i.e., SEM images can reveal presence of larger domains. We obtain an average domain size of ∼0.1 μm on Liq‐Cu and ∼0.2 μm on SC‐Cu. Overall, the GID results further demonstrate that the catalyst phase impacts in‐plane ordering and crystallite size, showing that SC‐Cu yields larger, more strongly oriented hBN domains, whereas Liq‐Cu produces a broad in‐plane orientation distribution and reduced coherence lengths. Notably, this contrasts with graphene on liquid Cu, where in situ GID revealed graphene BRs with resolution‐limited widths (crystallite sizes >10 μm) and no $q_{z}$‐dependent broadening, indicative of highly ordered, weakly corrugated monolayers [19, 29].

### 3.4 Interfacial Electron Density and Adsorption Height

**FIGURE 5:**
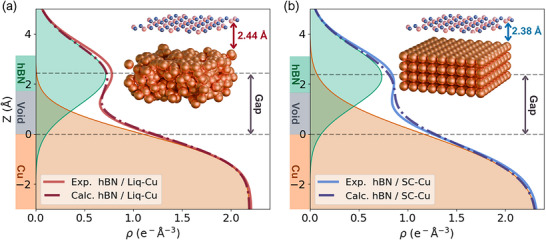
Electron density profiles along the surface normal (axis *Z*) for hBN on (a) Liq‐Cu and (b) SC‐Cu as determined experimentally (solid lines) and from atomistic simulations (dash‐dotted lines). The axis *Z* is referenced to the inflection point of the Cu electron density profile (*Z* = 0 Å), according to our definition of the “adsorption height” (see text). The sketches illustrate the interlayer spacing between hBN on Liq‐Cu and SC‐Cu.

To bridge the substrate‐phase‐dependent growth behavior to microscopic interface properties, we quantify the hBN/Cu adsorption geometry under operando conditions. We extract electron‐density profiles (ρ(Z)) normal to the interface by fitting a slab‐based model to the in situ XRR curves, focusing on the best‐defined reference cases of bare Cu and the Stage‐I (monolayer‐level) states (“Run I” on Liq‐Cu and “Run VI” on SC‐Cu in Figure 3). In the model, Cu is treated as a semi‐infinite substrate and hBN as an atomically thin slab (Figure 5), where the electron densities are assigned according to the respective temperature‐dependent atomic densities. Roughness parameters σ capture the interfacial broadening (modeled via error‐function smoothing) and are refined jointly with the Cu‐hBN separation distance during fitting (fit parameters are summarized in Table S4). The fitted roughness values indicate a narrow interfacial broadening on both substrates ($\sigma_{\mathrm{hBN}}\approx \sigma_{\mathrm{Cu}}=1.0-1.2\;Å$), consistent with a smooth surface and well‐defined monolayer. These roughness values are comparable to prior XRR‐based roughness values reported for graphene on Cu under similar conditions [19, 27, 69], as well as reports of hBN grown on other smooth substrates, but are substantially smaller than the interfacial broadening typically inferred for hBN grown on polycrystalline Cu [16, 76, 77, 78].

From the fitted ρ(Z) profiles (solid lines in Figure 5), we define the interfacial separation $d_{\mathrm{gap}}$ (or adsorption height) as the distance between the inflection point of the Cu electron‐density slab and the center of the hBN slab. This yields $d_{\mathrm{gap}}^{\mathrm{XRR}}=2.44\pm 0.06\;Å$ for hBN/Liq‐Cu and $d_{\mathrm{gap}}^{\mathrm{XRR}}=2.38\pm 0.05\;Å$ for hBN/SC‐Cu, where uncertainties represent the root‐mean‐square deviation across independent experiments. The smaller $d_{\mathrm{gap}}$ on SC‐Cu indicates a modestly stronger effective hBN‐Cu interaction compared to Liq‐Cu [27, 79]. Notably, this Cu substrate‐phase sensitivity contrasts with graphene, for which simulations found essentially identical adsorption heights on SCCu and Liq‐Cu, indicating adsorption dominated by van der Waals interactions [27]. These simulations were in quantitative agreement with experimental measurements for graphene on Liq‐Cu (cf. Table 2), which report an overall smaller adsorption height of $d_{\mathrm{gap}}=2.20\pm 0.10\;Å$ [19, 27, 69].

**TABLE 2:** Inflection‐point gap values for hBN (this work) and graphene [27], on Liq‐Cu and SC‐Cu, computed using ML‐potential molecular dynamics ($d_{\mathrm{gap}}^{\mathrm{MD}}$) and determined experimentally by XRR ($d_{\mathrm{gap}}^{\mathrm{XRR}}$).

	Liq‐Cu		SC‐Cu	
	$d_{\mathrm{gap}}^{\mathrm{XRR}}$ [Å]	$d_{\mathrm{gap}}^{\mathrm{MD}}$ [Å]	$d_{\mathrm{gap}}^{\mathrm{XRR}}$ [Å]	$d_{\mathrm{gap}}^{\mathrm{MD}}$ [Å]
Graphene	2.20	2.11	—	2.13
hBN	2.44	2.35	2.38	2.29

### 3.5 hBN/Cu Interfacial Interactions from Atomistic Simulations

To elucidate the microscopic origin of the hBN/Cu interaction and enable a direct comparison with graphene, we performed large‐scale MD simulations. These simulations were driven by accurately trained ML interatomic potentials, which enable nanosecond timescales to accurately sample hBN/Liq‐Cu and sufficiently large supercells to minimize artificial strain for hBN/SC‐Cu models (see Methods and Supporting Information for details).

The simulations reproduce the experimental electron‐density profiles closely (compare dashed and solid lines in Figure 5) and recover the substrate‐phase trend in adsorption geometry. Specifically, we obtain $d_{\mathrm{gap}}^{\mathrm{MD}}=2.35\;Å$ on Liq‐Cu and $d_{\mathrm{gap}}^{\mathrm{MD}}=2.29\;Å$ on SC‐Cu for hBN, using the same definition of $d_{\mathrm{gap}}$ as in the XRR analysis (see Table 2 and Table S8). For graphene, the corresponding values are $d_{\mathrm{gap}}^{\mathrm{MD}}=2.11\;Å$ on Liq‐Cu and $d_{\mathrm{gap}}^{\mathrm{MD}}=2.13\;Å$ on SC‐Cu (see Table 2 and Table S9), consistent with previous simulations and the experimental graphene value on Liq‐Cu ($d_{\mathrm{gap}}=2.20\pm 0.10\;Å$) [27]. Across systems, we observe only a small systematic underestimation of ∼0.1 Å compared to experimental values, in line with reported systematic deviations for simulated hBN adsorption heights on close‐packed metal surfaces [27, 80]. Importantly, whereas graphene shows, at best, only a very weak inverse phase dependence of $d_{\mathrm{gap}}$, hBN exhibits a clearly reduced $d_{\mathrm{gap}}$ on SC‐Cu. This trend suggests that the hBN/Cu interactions are more directional in character, which stabilizes the interface in the case of the solid substrate.

**FIGURE 6:**
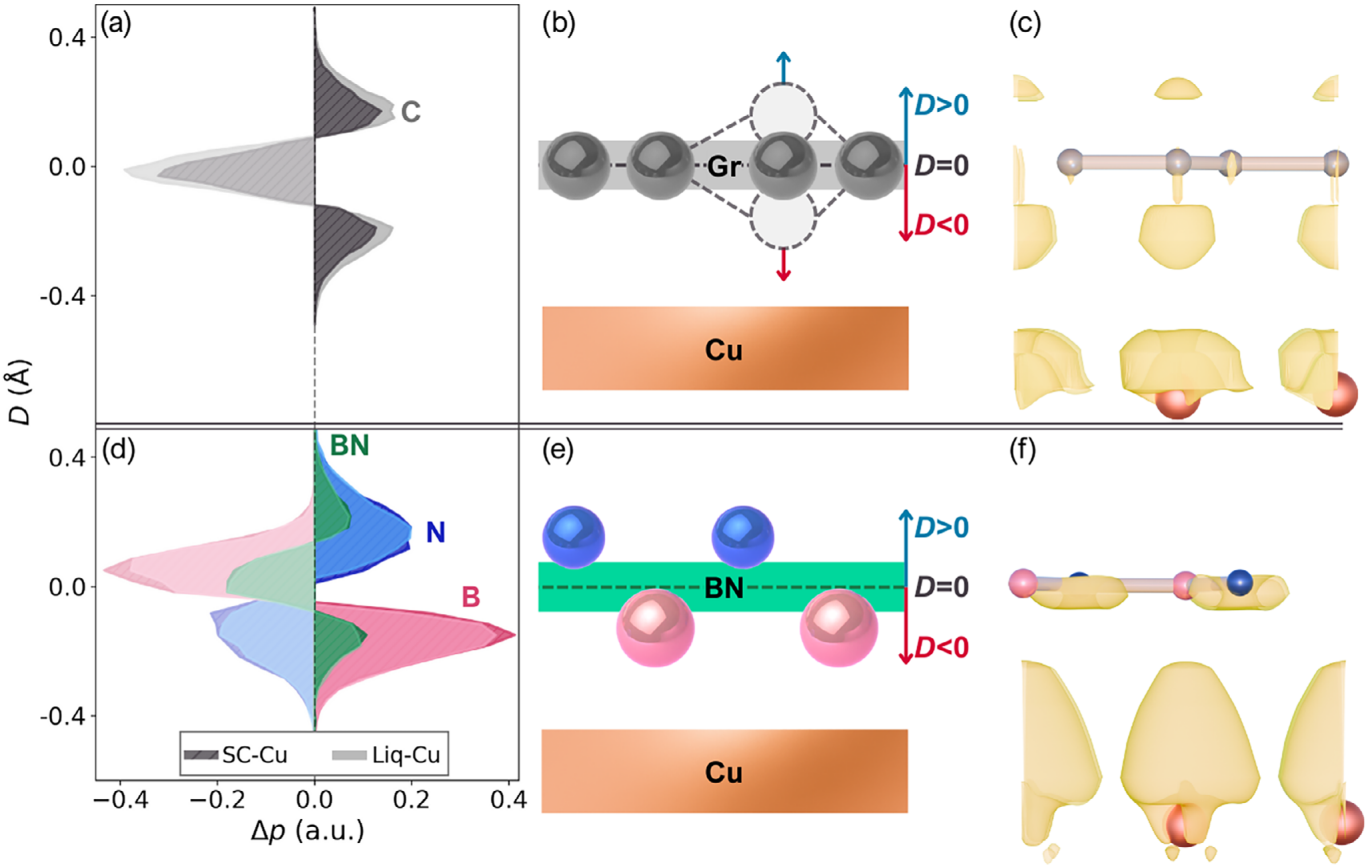
(a,d) Relative out‐of‐plane deformation for C/B/N atoms in Gr or BN during MD simulations. D (vertical axis) is the signed deformation distance defined as the distance between an atom and the plane formed by its three neighbors. The horizontal axis Δp represents the difference between the distributions of D for Gr and BN on SC‐Cu and Liq‐Cu slabs with respect to free‐standing equivalents (compare Table S7) (b,e) Schematic illustrations of the interfacial geometry. Negative D values indicate atoms displaced toward the Cu slab (below the Gr/BN sheet) and positive toward vacuum (above the Gr/BN sheet). (c,f) Electron density difference plots for Gr/Cu(111) and hBN/Cu(111) with respect to free‐standing 2DMs and bare Cu(111) in our minimal model (see text). Only positive isosurfaces are presented at a level of 0.001 Å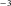 in both plots. Atomic color scheme: Cu (orange), C (gray), B (pink), N (blue).

The directional character of the hBN/Cu interactions compared to graphene/Cu is corroborated by qualitatively different interfacial corrugation motifs as revealed in the MD simulations (Figure 6a,b,d,e). hBN develops a pronounced asymmetric out‐of‐plane deformation, where B atoms preferentially displace toward the Cu surface while N atoms displace away from it, yielding a net “drag” of the sheet toward the substrate (Figure 6d,e; see also Table S10). This directionality is observed on both catalysts but is slightly enhanced on SC‐Cu (compare dark vs. light distributions in Figure 6d), consistent with the smaller separation distance and a more directional interaction component. In contrast, graphene exhibits only weak, largely symmetric corrugation (Figure 6a,b), in line with an interaction dominated by dispersion. Within this picture, graphene can interact marginally more strongly with Liq‐Cu, where the configurational freedom of the liquid allows, on average, more Cu atoms to closely approach the carbon lattice, which explains the slightly smaller gap distance compared to SC‐Cu (Figure 6a).

To probe the electronic origin of this directionality, we performed complementary DFT calculations on minimal 2DM/Cu(111) model interfaces (see Supporting Information for details), and analyzed the interfacial charge redistribution. From these very idealized calculations, we evaluate the interfacial charge transfer using a Hirshfeld charge analysis [81] (Table S11), which serves as a rough proxy for electronic hybridization. Both systems exhibit net charge transfer from Cu to the adsorbates, which is larger for hBN ( −0.02,e transferred predominantly to B) than for graphene ( −0.007,e per C), consistent with a stronger hybridization contribution in the former. Charge‐density‐difference maps further show electron accumulation in the interfacial B‐Cu region for hBN/Cu(111), producing a covalent‐like directional density feature that is absent for graphene/Cu(111) (Figure 6c,f). While the overall binding remains van der Waals‐dominated (as confirmed by an electron localization function analysis), these electronic signatures support an additional hybridization‐driven component for hBN that rationalizes its smaller adsorption height on SC‐Cu and the material‐specific substrate‐phase dependence observed experimentally.

## 4 Implications for High‐Quality Growth

The working assumption tested here for LMCats is that an atomically smooth, isotropic, and self‐healing liquid surface should inherently favor high‐quality two‐dimensional growth. This expectation is largely shaped by graphene, for which Liq‐Cu enables the formation of highly crystalline, strictly monolayer films via the merging of self‐aligned, up to millimeter‐sized single‐crystal flakes. In contrast, graphene growth on SC‐Cu is not only known to relay substrate defects to the overlayer but has an inherently higher nucleation density of immobile, misoriented domains, which result in increased polycrystallinity [18, 20]. Our operando observations demonstrate that hBN does not simply “inherit” this graphene paradigm under otherwise comparable near‐ambient‐pressure conditions. Instead, hBN grown on Liq‐Cu exhibits powder‐like diffraction signatures, indicative of a broad in‐plane orientation distribution and limited orientational self‐organization on the liquid surface, and evolves toward multilayer and three‐dimensional outgrowth. On re‐solidified SC‐Cu, by contrast, hBN remains predominantly monolayer‐limited over a broad precursor‐exposure window and develops somewhat larger domains with a narrower in‐plane orientation distribution. These findings cannot be attributed to the small difference in growth temperatures of ∼15 K (see Supporting Information) and instead challenge the presumed universality of the liquid‐catalyst advantage and highlight a pronounced material‐specific response to the catalyst phase.

To understand why the catalyst phase exerts such a strongly material‐dependent influence, we turn to microscopic insight obtained from our operando XRR and complementary atomistic simulations. A key distinction relative to graphene is that the hBN/Cu interface exhibits directional and asymmetric interfacial distortions accompanied by charge redistribution [33], rather than an interaction dominated almost exclusively by dispersion forces. Such directional bonding contributions can be stabilized more effectively on a structurally ordered SC‐Cu surface than on a dynamically disordered liquid interface, providing a microscopic basis for the contrasting behavior with graphene, for which such contributions are negligible. Within this picture, interfacial stabilization emerges as an important microscopic parameter, influencing growth quality. This interpretation is consistent with classical thin‐film growth modes, where stronger wetting and adhesion favor layer‐by‐layer growth, while weaker interfacial stabilization promotes three‐dimensional island formation [82, 83], In this context, the separation distance (or adsorption height) provides a physically meaningful descriptor governing interfacial stabilization, wetting, and layer adhesion [31, 32, 33, 34]. Indeed, across the systems studied here, we observe a qualitative inverse correlation between adsorption height and film quality, assessed in terms of crystallinity and monolayer termination, following the trend graphene/Liq‐Cu ≥ graphene/SC‐Cu > hBN/SC‐Cu > hBN/Liq‐Cu.

This interfacial‐stabilization perspective naturally complements the established rationalization of graphene CVD on Cu as a surface‐limited process, where carbon dissolves only weakly in the catalyst and the first graphene layer passivates active sites for precursor decomposition, thereby suppressing multilayer formation [84]. For hBN, however, the dual‐species chemistry introduces additional asymmetry [85]. Boron‐containing species can dissolve into the Cu catalyst, whereas nitrogen is supplied primarily through surface‐mediated reactions [23]. The substantially higher solubility of B in molten Cu compared to solid Cu [86], combined with enhanced transport and mixing in the liquid phase, sustains continued delivery of growth species to the interface. The pronounced grain‐boundary network of hBN on Liq‐Cu further promotes multilayer and three‐dimensional outgrowth even after the first hBN layer has formed. On a solid SC‐Cu surface, by contrast, reduced B bulk‐enrichment combined with stronger interfacial stabilization favors effective surface passivation and supports a monolayer‐limited growth regime. Together, these considerations suggest that precursor solubility and associated species supply, on the one hand, and interfacial stabilization, on the other, act as complementary factors in governing such dissolution‐segregation processes and, consequently, mono‐ vs. multilayer growth. In practical terms, these insights motivate design strategies for hBN CVD on Cu; specifically, controlling precursor exposure to remain within a monolayer‐growth window when self‐termination is desired, and selecting catalyst phase and surface crystallinity to tune the balance between interfacial passivation and multilayer or three‐dimensional nucleation.

## 5 Conclusions

In this work, we combine operando optical microscopy with in situ synchrotron surface‐sensitive GID and XRR, complemented by ex situ spectroscopy/microscopy and atomistic simulations, to resolve growth‐stage signatures and interfacial structure of hBN during CVD growth on molten and re‐solidified single‐crystal copper under near‐ambient‐pressure conditions. From this wealth of complementary methods, a central advance is the reliable acquisition of in situ reflectivity curves with monolayer sensitivity and diffraction maps resolving hBN 2D Bragg rods during growth on both molten and re‐solidified copper. These measurements remain rare under near‐ambient‐pressure CVD and are enabled here by a high‐brilliance synchrotron source and a curvature‐aware reflectivity reconstruction tailored to liquid‐metal surfaces.

Across our operando observables and SEM, hBN exhibits a pronounced substrate‐phase dependence. On molten copper, a monolayer‐level stage is followed readily by multilayer/3D outgrowth and a powder‐like in‐plane orientation distribution, whereas on re‐solidified copper the growth remains predominantly monolayer‐limited over a broad precursor‐exposure window. GID yields in‐plane lattice parameters of $a_{1}=2.514\pm 0.006\;Å$ (Liq‐Cu) and $a_{1}=2.510\pm 0.008\;Å$ (SC‐Cu), and indicates a two‐fold increase in lateral grain size from ∼0.1 μm to ∼0.2 μm on the re‐solidified substrate. From XRR fits of the monolayer‐level stage, we extract a reproducible interfacial separation difference: $d_{\mathrm{gap}}^{\mathrm{XRR}}=2.44\pm 0.06\;Å$ on molten copper vs. $d_{\mathrm{gap}}^{\mathrm{XRR}}=2.38\pm 0.05\;Å$ on re‐solidified copper. ML‐accelerated molecular dynamics simulations reproduce this trend ($d_{\mathrm{gap}}^{\mathrm{MD}}=2.35\;Å$ and 2.29 Å, respectively) and, together with DFT‐based electronic‐structure analysis, support a directional, sublattice‐asymmetric interaction at the hBN/Cu interface that differs from the largely dispersion‐dominated graphene/Cu case.

Taken together, these results demonstrate that the frequently assumed “liquid‐catalyst advantage” is not universal across 2DMs. Under comparable conditions, Liq‐Cu promotes multilayer/3D outgrowth of hBN, whereas re‐solidified SC‐Cu stabilizes a predominantly monolayer‐limited regime with higher film quality and larger crystalline domains. More generally, our findings identify interfacial stabilization, quantified here through the hBN‐Cu adsorption height as a descriptor for associated layer adhesion, as a potentially important microscopic factor influencing mono‐ vs. multilayer growth, complementary to macroscopic factors such as precursor solubility and the associated dissolution‐segregation pathways.

## Author Contributions

N.Gh. performed the experiments, analyzed and interpreted the data, and wrote the original draft of the manuscript. V.R. led the project and, together with O.K., conceived and supervised the research. V.R. contributed to experimental design, experiments, data analysis, interpretation, and manuscript writing and editing. H.G. led the computational work, developed the atomistic/DFT model, performed the simulations, and contributed to mechanism analysis, data interpretation, and manuscript writing and editing. E.B. and T.S. assisted with experiments, data analysis, and manuscript proofreading. M.K.S. assisted with experiments. B.C. performed and analyzed AFM measurements. I.S. performed SEM measurements. N.G. carried out XPS measurements and analysis. A.C.M. performed Raman measurements and analysis. M.J. assisted with experiments, data analysis, and manuscript proofreading. G.R. contributed research guidance and manuscript proofreading. A.D. coordinated the 2D‐ENGINE project. K.R. contributed to manuscript proofreading. H.H.H. supervised the computational work and contributed to data interpretation and manuscript writing and editing. I.M.N.G. contributed to research guidance and manuscript proofreading. All authors approved the final version of the manuscript.

## Supporting information


**Supporting File 1**: advs76354‐sup‐0001‐SuppMat.pdf.

**Supporting File 2**: Rad‐OM video of growth on Liq‐Cu.

**Supporting File 3**: Rad‐OM video of hBN growth on SC‐Cu.

**Supporting File 4**: GID detector images of hBN [11] BRs on Liq‐Cu.

**Supporting File 5**: GID detector images of hBN [11] BRs on SC‐Cu.

## Data Availability

The raw X‐ray data underlying this study are openly available in the European Synchrotron Radiation Facility Data Portal (https://doi.esrf.fr/10.15151/ESRF‐ES‐1226443495, https://doi.esrf.fr/10.15151/ESRF‐ES‐1950221244, https://doi.esrf.fr/10.15151/ESRF‐ES‐2166885235). Derived data supporting the findings of this study are available from the corresponding author on request. The data treatment software developed for this study, FishingROD, is openly available (https://github.com/Nikoo‐Ghn/FishingROD).
