# Supplementary material for: Hexagonal Boron Nitride on Liquid and Single‐Crystal Copper: Operando X‐Ray and Atomistic Insights into Growth and Interfacial Structure
Source: Adv Sci (Weinh). 2026 Jul 20;13(53):e76354. doi: 10.1002/advs.76354 (PMC13383709; doi:10.1002/advs.76354)
Supplement: Supplementary file 1 [file ADVS-13-e76354-s001.pdf]

Supporting Information : Hexagonal Boron Nitride on Liquid and Single-Crystal Copper: Operando X-Ray and Atomistic Insights into Growth and Interfacial Structure

Nikoo Ghanadan,[†,‡] Valentina Rein,[*,†] Hao Gao,[¶] Egor Bersenev,[†,§] Thomas Sarrazin,[†,∥] Manmeet Kaur Sodhi,[⊥,∥] Bárbara Canto,[#] Irina Snigireva,[†] Nicolas Gauthier,[∥,@] Anastasios C. Manikas,[△,▽] Costas Galiotis,[△,▽] Maciej Jankowski,[†] Gilles Renaud,[⊥,∥] Athanasios Dimoulas,[††] Karsten Reuter,[¶] Oleg Konovalov,[†] Hendrik H. Heenen,[*,¶] and Irene M.N. Groot[*,‡]

†*The European Synchrotron Radiation Facility (ESRF), Grenoble, France*

‡*Leiden Institute of Chemistry, Leiden University, Einsteinweg 55, 2333 CC Leiden, the Netherlands*

¶*Fritz-Haber-Institut der Max-Planck-Gesellschaft, Faradayweg 4-6, D-14195 Berlin, Germany*

§*School of Chemistry, University of Bristol, Bristol, United Kingdom*

∥*Université Grenoble Alpes, Grenoble, France*

⊥*CEA, IRIG/MEM/NRS, Grenoble, France*

#*AMO GmbH, Advanced Microelectronic Center Aachen, Otto-Blumenthal-Str. 25, 52074 Aachen, Germany*

@*CEA, LETI, Grenoble, France*

△*Institute of Chemical Engineering Sciences, Foundation of Research and Technology Hellas, Patras, Greece*

▽*Department of Chemical Engineering, University of Patras, Patras, Greece*

††*National Centre for Scientific Research Demokritos, Agia Paraskevi, Greece*

E-mail: valentina.rein@esrf.fr; heenen@fhi.mpg.de; i.m.n.groot@lic.leidenuniv.nl

Materials.

Copper foil from *Advent Research Materials* (Product No.: CU133419), tungsten support (from *Metel*), solution of 1:3 H_2O_2 : NH_4OH, from *Sigma-Aldrich* (Product No.: H1009 and 221228 respectively). H_2 (from *Air Liquide*), Ar (*Air Liquide*) Ammonia borane (AB) powder (*Sigma-Aldrich*, Product No. 682098) was used as the precursor and mixed in a 1:10 matrix with boron nitride (BN) powder (Sigma-Aldrich, Product No. 255475) to suppress AB foaming.[1]

Sample Preparation and CVD Setup.

The original configuration of the LMCat reactor was tailored specifically for the growth of graphene.[2] In order to adapt the reactor for hBN growth, the gas inlet was modified such that it passed through a cell equipped with a PID-controlled heating element, holding the powder precursor, ammonia borane (AB). When exposed to heat, AB powder tends to polymerize and foam, a process that is inherently unpredictable due to the irregular formation and propagation of polymer seeds, which can lead to inconsistent results across experiments. To mitigate this issue, as suggested by Babenko et al.,[1] boron nitride (BN) powder, was mixed at an AB:BN ratio of 1:10, to separate the granules and suppress foaming. To ensure uniform powder distribution, the AB:BN mix was ground using a mortar and pestle. For each experiment, roughly 100 mg of this mix was placed inside our cell.

The substrates were prepared according to the following procedure: two ⌀ 15 mm, 50 µm-thick circular sheets of copper were placed on a tungsten support, chemically etched at its center using base piranha solution (1:3 solution of H_2O_2:NH_4OH), to reduce its effective contact angle with the liquid Cu.

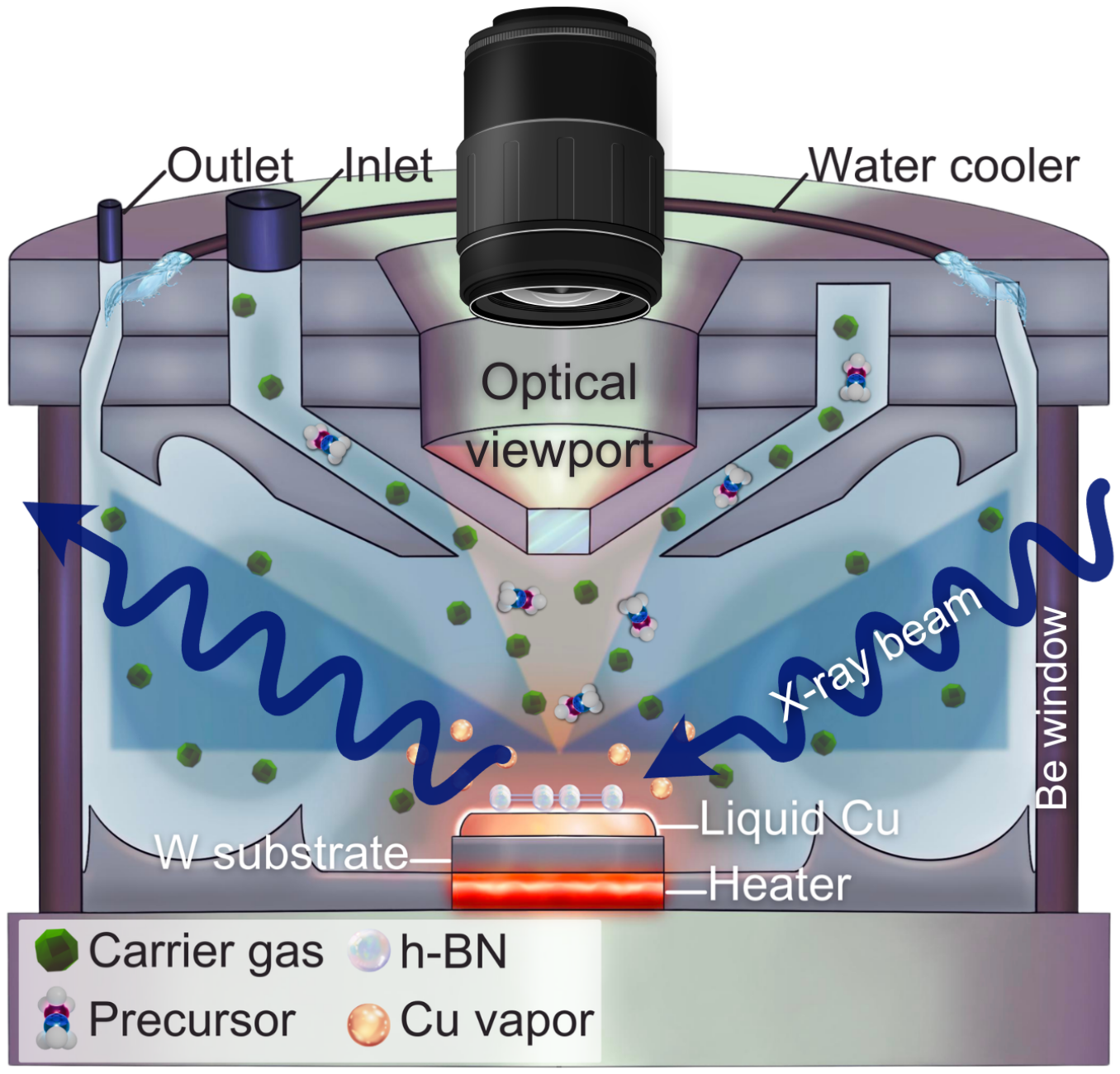

Figure S1: Schematic illustration of the LMCat CVD reactor.[2]

The CVD LMCat reactor allows precise control over base pressure, temperature, and uniform gas flow.[2] The reactor design enables real-time monitoring of 2DMs growth by featuring a beryllium wall for minimal X-ray absorption and a quartz window for Rad-OM sample observation while protecting them from excessive metal deposition.

The growth conditions for all samples were maintained at 200 mbar pressure, ∼1100°C reactor temperature, and a carrier gas flow of 200 sccm Ar and 20 sccm H_2. Key variables, such as growth duration, AB cell temperature, and argon flow through the powder cell, were varied and closely monitored across all samples.

Remark on Growth Temperature Differences.

We note that the origin of the observed inversion in growth behavior with respect to graphene cannot be attributed to a small difference in growth temperatures (a residual offset of only ∼15 K, i.e., ∼1% of the absolute growth temperature). In fact, hBN-on-Cu literature generally reports that increasing growth temperature favors improved monolayer quality, lower nucleation density, and/or larger domain size, whereas in our experiments, the slightly higher-temperature

Liq-Cu condition shows the opposite trend.[3–5] In addition, a simple Eyring-type estimate using representative activation barriers indicates that increasing the temperature from 1355 to 1370 K changes the relative competition between growth and nucleation pathways only weakly (on the order of 5-10%), supporting the conclusion that the dominant difference arises from the catalyst phase.[6,7]

XPS.

We used XPS to verify the chemical composition of the as-grown layers. Figure S2 (b, c) shows high-resolution spectra of the N 1s and B 1s core levels. The characteristic peaks found at 190.4 eV (B 1s) and 397.9 eV (N 1s) agree well with literature values for stoichiometric hBN.[8,9] The pseudo Voigt (Gaussian/Lorentzian Product Form GL(35)) fit of the peaks yielded an atomic ratio of N:B = 0.98, indicating a stoichiometric hBN.

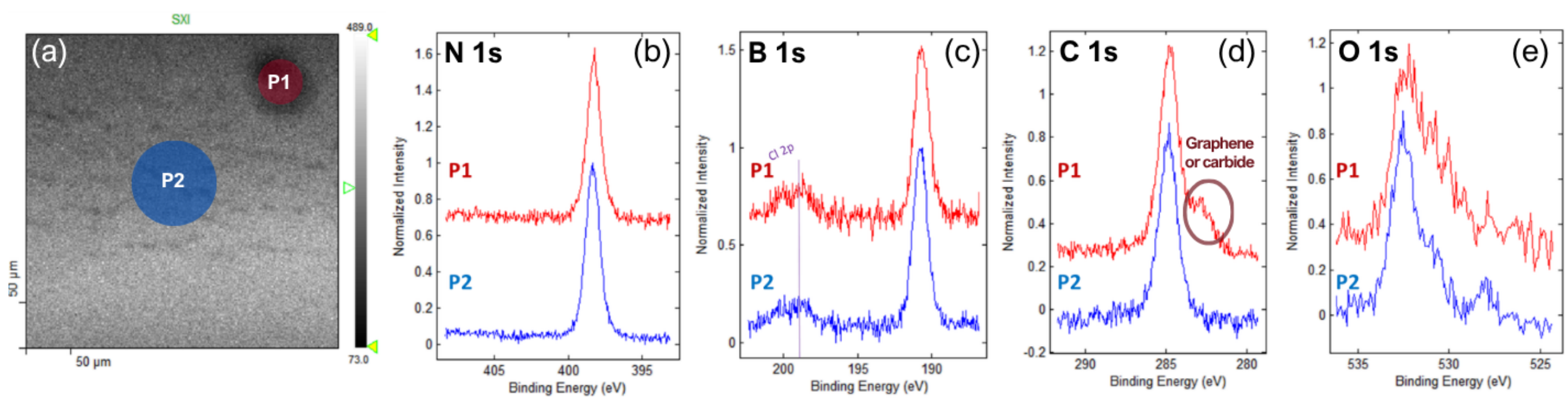

Figure S2: (a) Scanning X-ray-induced secondary electron (SXI) image of the hBN sample, with the analyzed regions labeled P1 and P2. (b-e) Core-level XPS spectra of the sample. Stoichiometric hBN was observed throughout the sample, with a [N]/[B] ratio of 0.98

In addition to B and N, contributions of carbon (>20 at.%) and oxygen (>5 at.%) were detected. Traces of chlorine were also detected. We note that XPS measurements were performed ex situ and are therefore sensitive to ambient exposure, handling, and the initial surface state of the Cu substrate. Carbon contamination is present on Cu already prior to growth, likely due to precursor contamination, and varies locally across the surface. In the present dataset, one localized small-area measurement point P1 shows substantially higher carbon content than the more representative regions, such as P2 (44 at.% vs 23 at.%). Oxygen and chlorine remain

minor components. The chlorine likely originates from the Cu substrate[10] or its prior handling. Notably, the B 1s and N 1s line shape and FWHM (∼1.1 eV) remain unchanged across the sampled areas, indicating a chemically consistent hBN phase despite the presence of ex situ surface contamination.

Raman Spectroscopy.

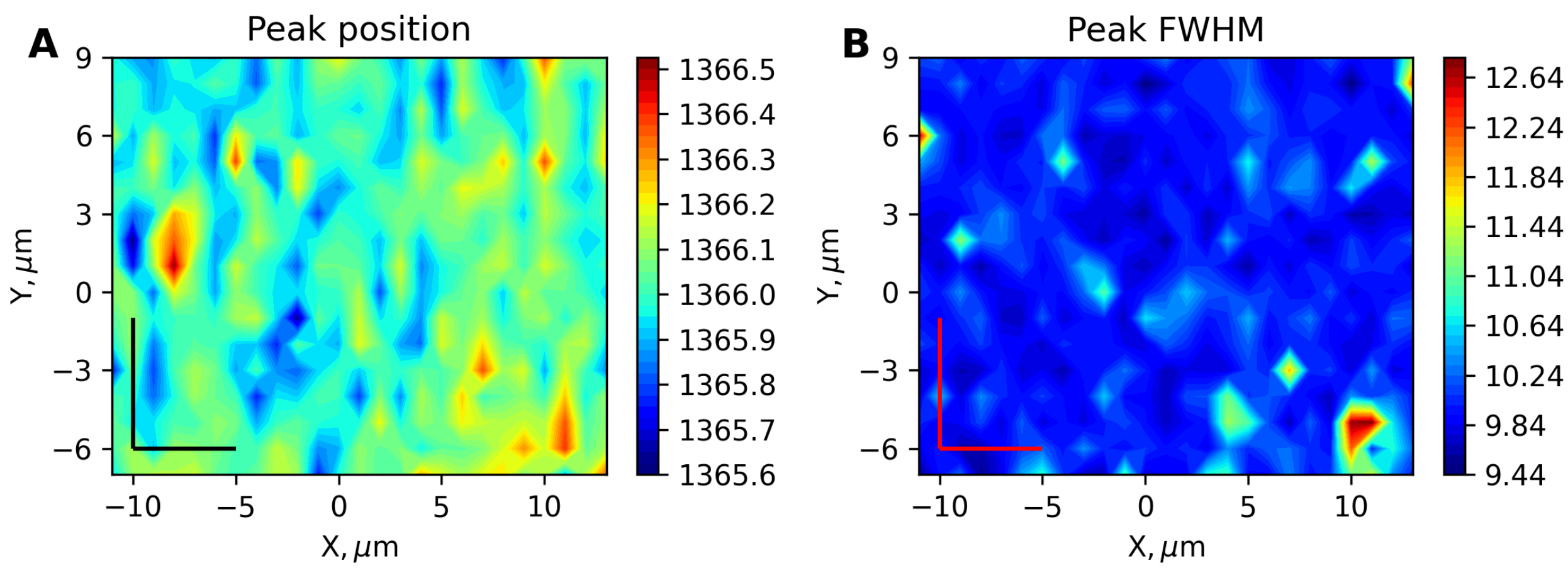

Figure S3: Raman mapping of an hBN region deposited on a Si/SiO_2 substrate: (A) Raman peak position (cm^{-1}) and (B) full width at half maximum (FWHM, cm^{-1}). Scale bars correspond to 5 μm in both directions.

AFM.

AFM measurements were performed to obtain information about the different layers of hBN grown on Cu. The Cu droplets tend to be curved, which makes AFM imaging and interpretation difficult. Therefore the samples had to be transferred to a flat substrate; here we used thermally grown 90 nm SiO_2/Si. After the transfer, samples were scanned in air at room temperature. The scanned area was 10×10 μm^2. The resulting images are not expected to represent the pristine growth-stage interface with the same fidelity as the in situ XRR data. Figure S4 shows representative AFM topography of transferred hBN grown on Liq-Cu (a), which exhibits more considerable height variation and greater overall topographic relief than hBN grown on SC-Cu (b), which is notably flatter. Both samples display triangular hBN domains as well as residual

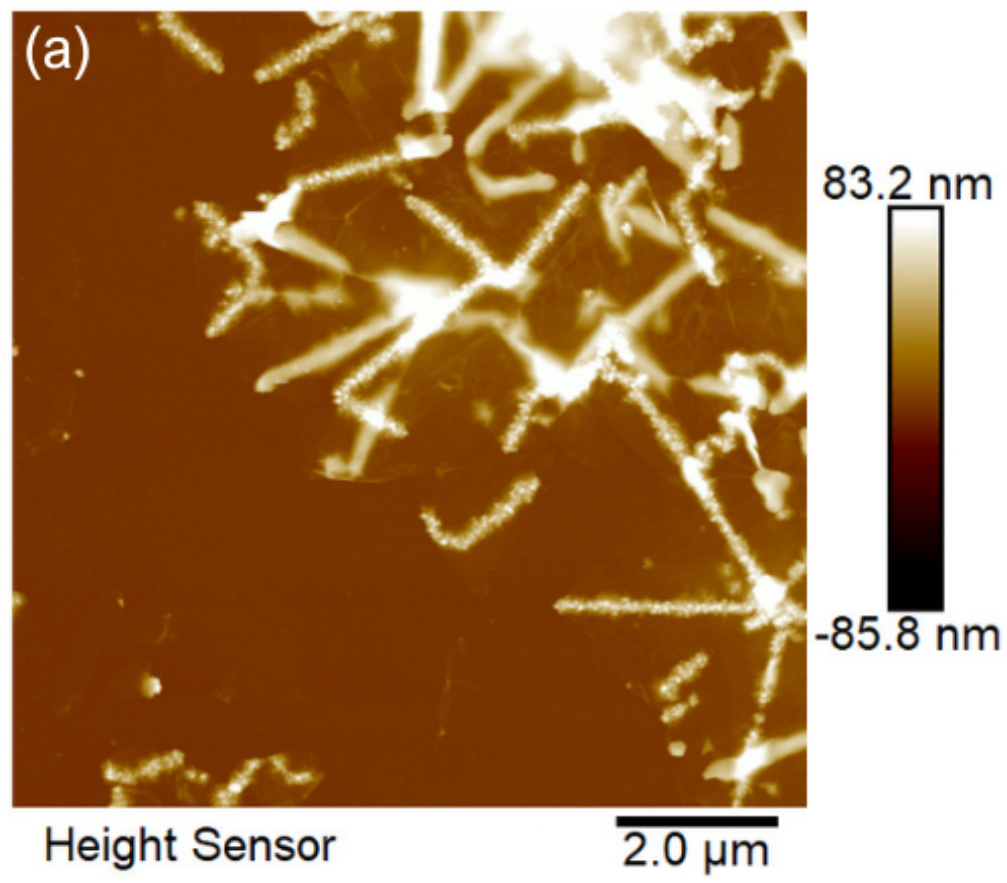

Figure S4: Representative AFM topography of transferred hBN grown on Liq-Cu (a) and SC-Cu (b). The height scales differ between panels for clarity.

PMMA from the transfer process as can be seen in Figure S4 (b) as a rough surface.

SEM.

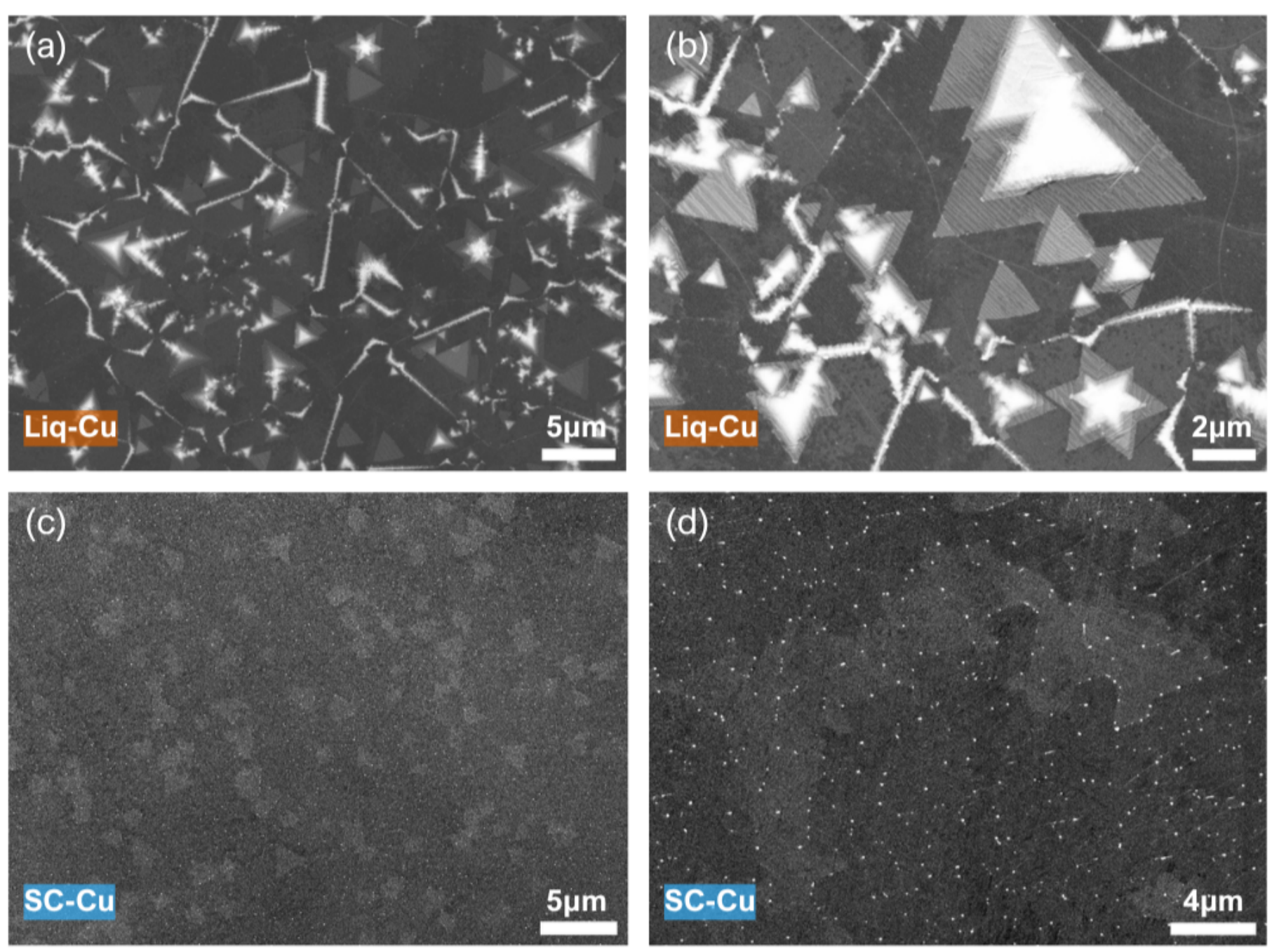

Figure S5: Representative SEM images of as-grown hBN on Liq-Cu (a,b) and SC-Cu (c,d). Bright areas correspond to thicker hBN overgrowth features or crystal stacks relative to the darker triangular regions. Images were acquired with the *Zeiss SUPRA 60VP* with a beam energy of 2.00 keV.

In Situ Optical Microscopy.

On Liq-Cu, optical images acquired during hBN growth exhibit discernible intensity variations that can be attributed to the formation of hBN (Figure 2, video S1). On SC-Cu however, the Rad-OM images (Figure S7 a-c, video S2) show only a small intensity drop upon monolayer formation. The image intensity signals shown in both Figures 2 (d) and S7 (d) were corrected for multiplicative thermal drift by fitting a polynomial regressor to reference images and temperature data acquired prior to growth, and normalizing the measured intensity accordingly. While this procedure reduces thermal noise, it does not fully eliminate it.

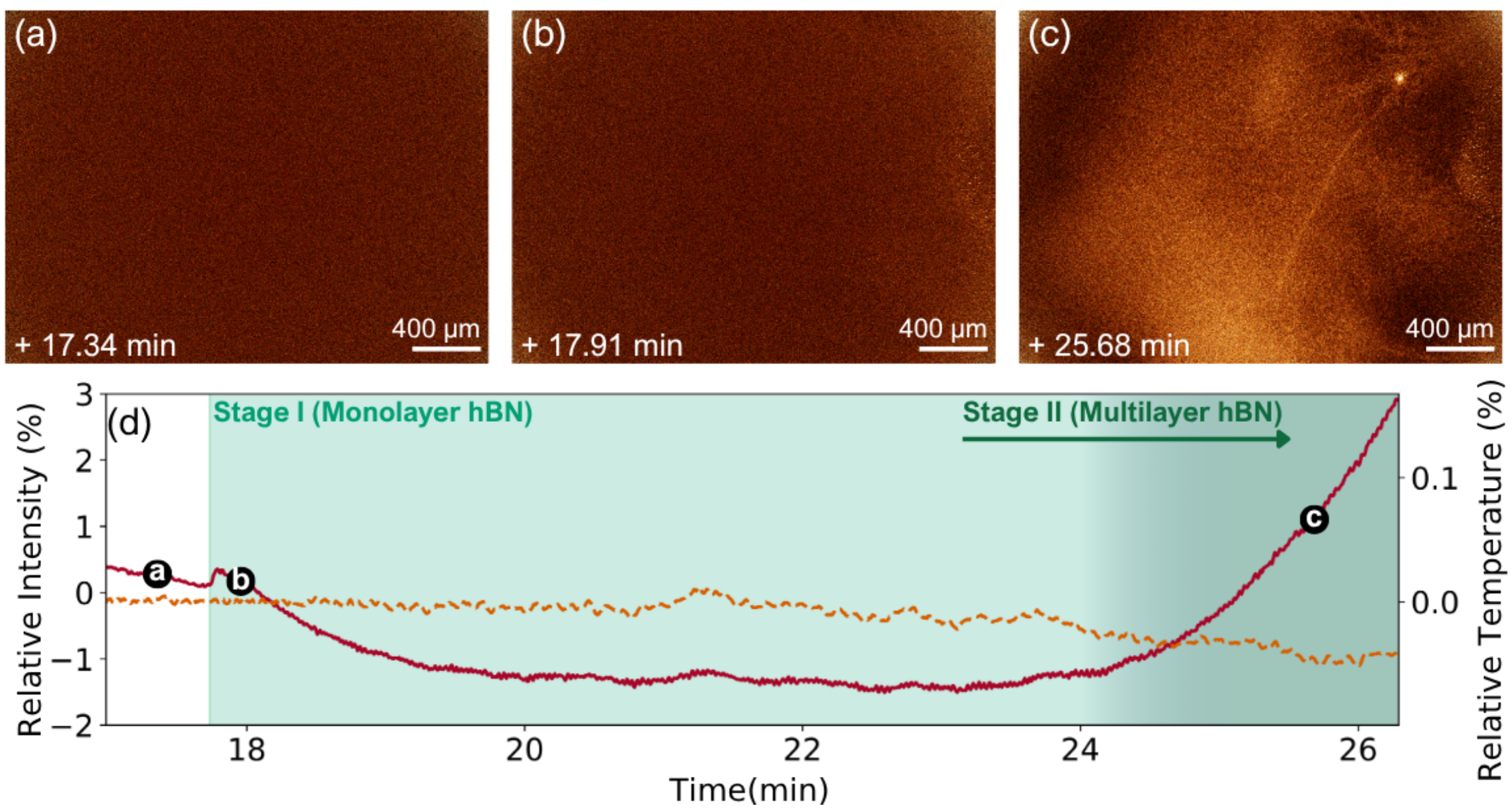

Figure S6: In situ Rad-OM measurements during growth: (a-c) False-color raw image (without averaging and Gaussian blurring) of hBN on Liq-Cu, showing (a) the bare Liq-Cu surface, (b) a monolayer hBN film with few 3D islands, (c) multilayer hBN. (d) Background-normalized average image intensity as a function of time (solid lines) and temperature variation (dashed lines). The intensity signal has been corrected for multiplicative thermal drift. The time point t = 0 corresponds to the moment at which the AB precursor cell temperature exceeds 60 °C. The shaded region (green) marks the time at which monolayer hBN is detected via X-ray characterization.

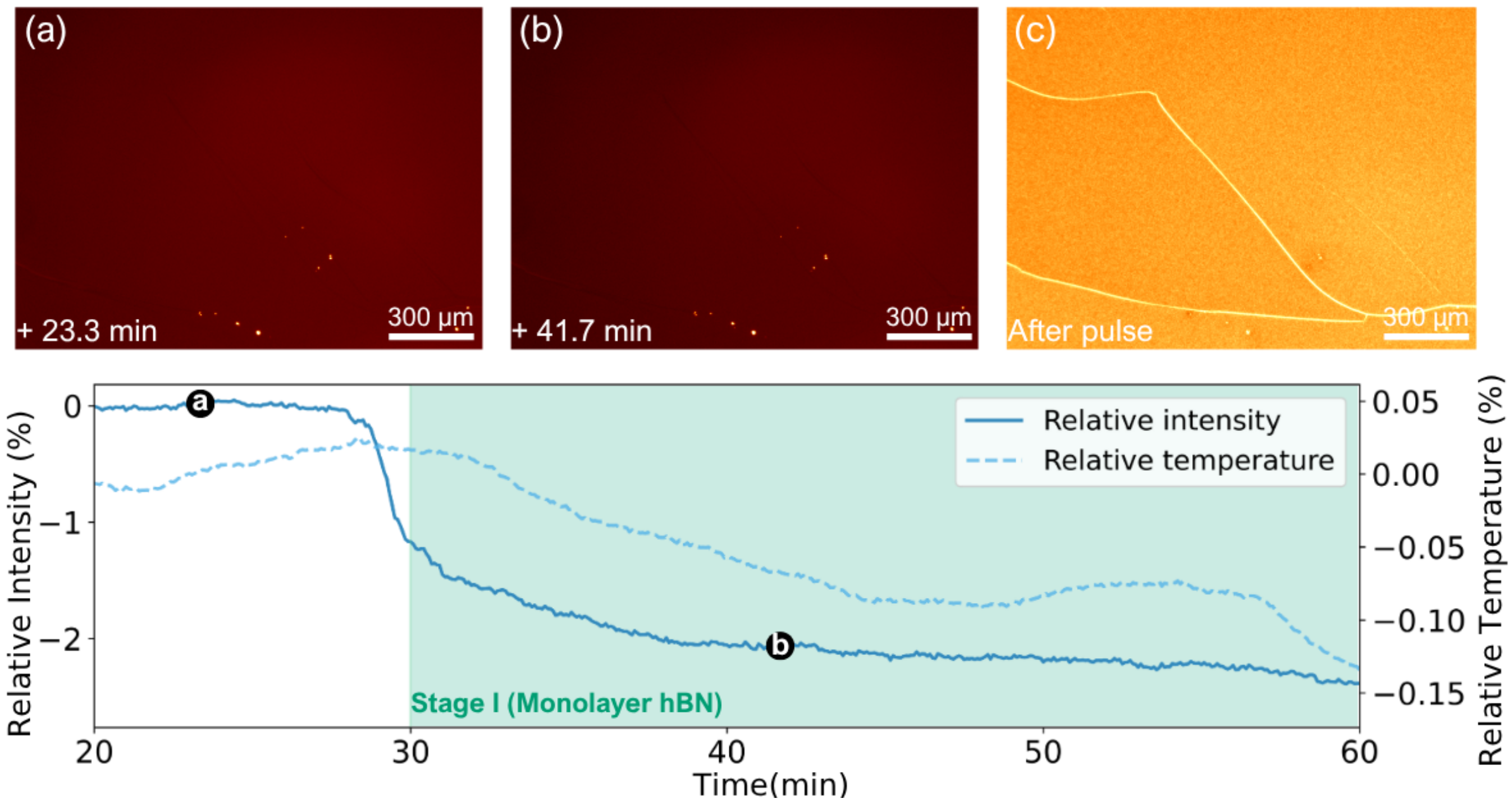

Figure S7: In situ Rad-OM measurements during growth: (a-c) False-color raw image (without background subtraction) of hBN on SC-Cu, showing (a) the bare SC-Cu surface, (b) a monolayer hBN film with few 3D islands, (c) multilayer growth induced by a high-flux precursor pulse. (d) Background-normalized average image intensity as a function of time (solid lines) and temperature variation (dashed lines). The intensity signal has been corrected for multiplicative thermal drift. The time point $t = 0$ corresponds to the moment at which the AB precursor cell temperature exceeds 60 °C. The shaded region (green) marks the time at which monolayer hBN is detected via X-ray characterization.

Precursor Exposure Metric Calculation for Ammonia Borane cell.

To compare the AB precursor exposure corresponding to the XRR scans in Figure 3, we define an operational metric based on the recorded AB-source temperature and gas-flow profiles. Because ammonia borane does not behave as a simple subliming precursor but undergoes temperature- and time-dependent decomposition, an absolute dose cannot be reliably defined. We therefore adopt a relative exposure metric that is internally consistent within our setup. As Antoine coefficients for AB are not available, we estimate an equilibrium vapor-pressure proxy using the integrated Clausius-Clapeyron[11] relation:

$$p(T) = p_{\mathrm{ref}} \exp\left[-\frac{\Delta H_{\mathrm{sub}}}{R}\left(\frac{1}{T_{AB}} - \frac{1}{T_{\mathrm{ref}}}\right)\right] \tag{1}$$

where T_{AB} is the source temperature for each run shown in figures S8 and S9, R = 8.314 J mol^{-1} K^{-1} is the gas constant, and ΔH_{sub} is the molar enthalpy of sublimation. Literature values for ΔH_{sub} vary substantially. Here we use ΔH_{sub} =76.0 kJ mol^{-1}, consistent with reported vapor pressure measurements of $\sim 10^{-4}$ Torr at 295 K.[12] Using p_{ref}=1.33×10^{-4} mbar and T_{ref}=295 K this yields:

$$p_{\mathrm{sat}}(T) = 1.33 \times 10^{-4} \exp\left[-9141\left(\frac{1}{T_{AB}} - \frac{1}{295}\right)\right] \quad [\mathrm{mbar}]. \tag{2}$$

To account for gas flow dilution, we define the instantaneous mole fraction:

$$\phi(t) = \frac{Q_{AB}(t)}{Q_{AB}(t) + Q_{\mathrm{Ar}} + Q_{\mathrm{H_2}}} \tag{3}$$

where Q_{Ar} =200 sccm and $Q_{\mathrm{H_2}}$=20 sccm and Q_{AB}=$Q_{\mathrm{Ar}} + Q_{\mathrm{H_2}}$ for growth runs on Liq-Cu and 5 sccm $\leq Q_{AB} \leq$ 50 sccm for growth runs on SC-Cu. This difference arises from the distinct growth dynamics on each substrate. On SC-Cu, a minimal precursor flux of 5-20 sccm was sufficient to form a monolayer. On Liq-Cu, however, routing the precursor through the high-flow main line (220 sccm) yielded better results than the dedicated low-flow line, making it the preferred configuration for this substrate. The effective AB partial pressure at the reactor can be written formally as:

$$p_{AB,\mathrm{eff}}(T,t) = \eta(Q_{\mathrm{cell}})\, p_{\mathrm{sat}}(T_{AB}(t))\, \phi(t) \quad [\mathrm{mbar}] \tag{4}$$

where η is an unknown flow- and configuration-dependent saturation efficiency of the AB source. This parameter accounts for possible under-saturation of the carrier gas inside the precursor cell and depends on factors such as gas residence time, source geometry, precursor history, and decomposition state. Because η was not independently calibrated, we do not assign numerical values to it and do not use Eq. 4 to determine an absolute AB partial pressure or molar dose. Instead, we use an onset-normalized exposure metric based only on the experimentally recorded source-temperature and flow profiles. In this metric, the precursor exposure is referenced to the experimentally observed growth-onset condition for each delivery configuration, so that unknown

configuration-dependent prefactors are absorbed into the normalization. The onset-normalized exposure time is defined as:

$$\tilde{D} = \int_{t_0}^{t_1} \frac{p_{\mathrm{sat}}(T_{AB}(t))\,\phi(t)}{p_{\mathrm{sat}}(T_{\mathrm{onset}})\,\phi_{\mathrm{onset}}}\,dt \quad [\mathrm{s}] \tag{5}$$

where T_{onset} and ϕ_{onset} correspond to the experimentally observed growth-onset conditions (ϕ_{onset} corresponds to the $Q_{\mathrm{AB}} = 220$ sccm and 1 sccm, and $T_{\mathrm{onset}} \simeq 85$ °C and $\simeq 60$ °C for the Liq-Cu and SC-Cu configurations respectively). By construction, the integrand equals unity at the growth-onset condition. Thus, $\tilde{D}$ has units of seconds and can be interpreted as an onset-equivalent exposure time. Remaining uncertainties arise from the AB decomposition chemistry, source-history effects, precursor depletion, and the unknown flow-dependent saturation efficiency,[1,13] and limit the metric to qualitative comparison within a given experimental configuration.

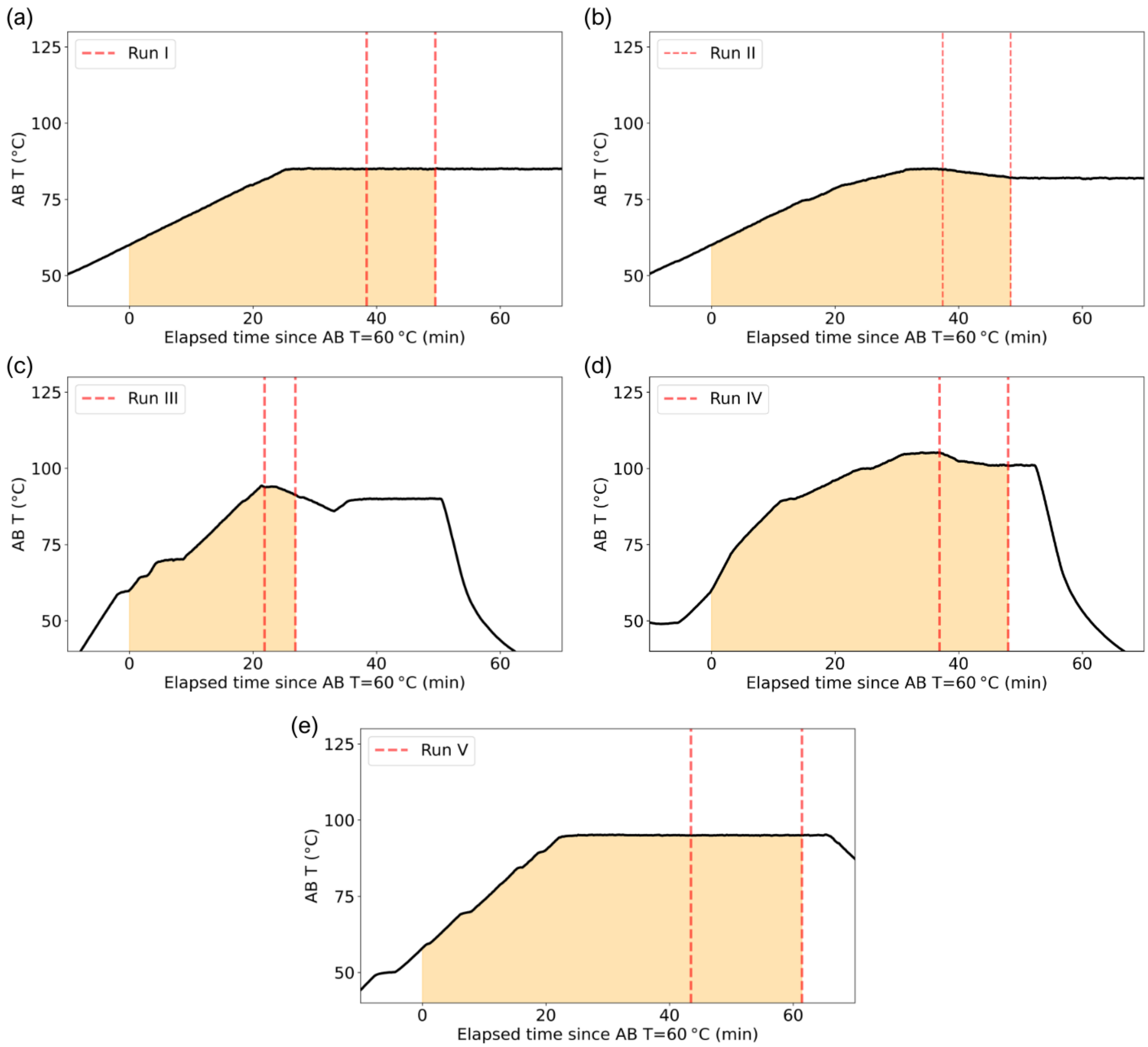

Figure S8: Growth conditions during hBN deposition for the XRR curves shown in Figure 3 (a). Each figure corresponds growth runs on to Liq-Cu. Dotted lines indicate the start and end points of the XRR scans. Time zero ($t = 0$) marks the moment the AB cell temperature goes above 60°C, taken as the onset of precursor exposure to the sample. The AB dose is defined as an integral for each scan.

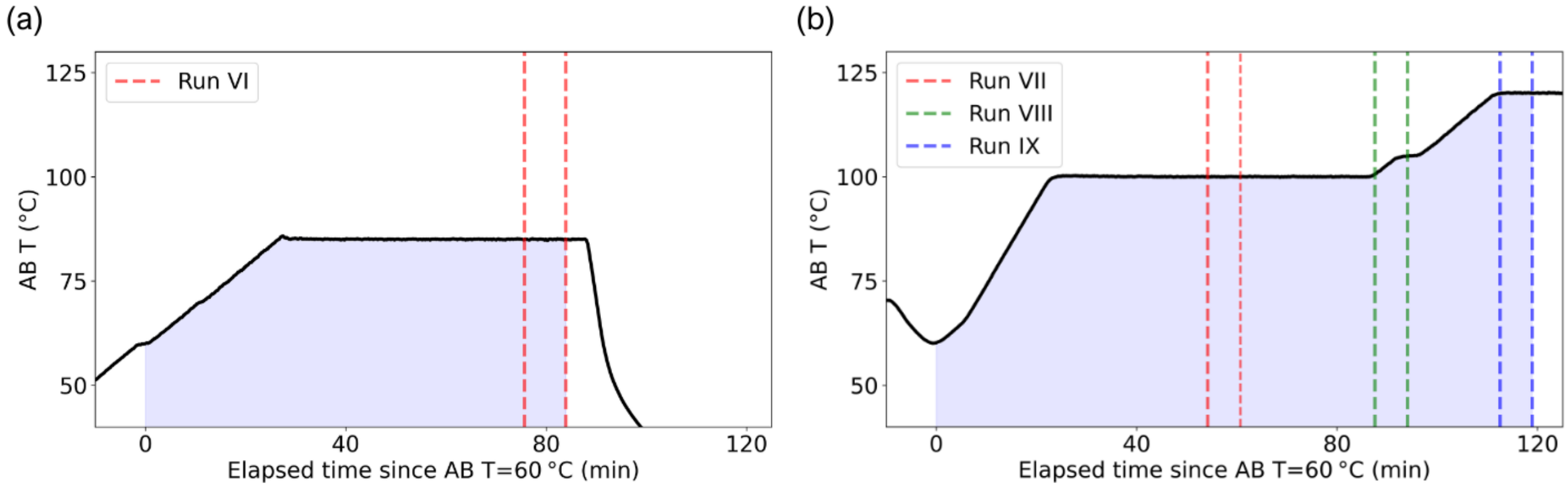

Figure S9: Growth conditions during hBN deposition for the XRR curves shown in Figure 3 (b). Each figure corresponds to growth runs on SC-Cu. Dotted lines indicate the start and end points of the XRR scans. Time zero ($t = 0$) marks the moment the AB cell temperature goes above 60°C, taken as the onset of precursor exposure to the sample. The AB dose is defined as an integral for each scan.

In Situ X-ray Reflectometry.

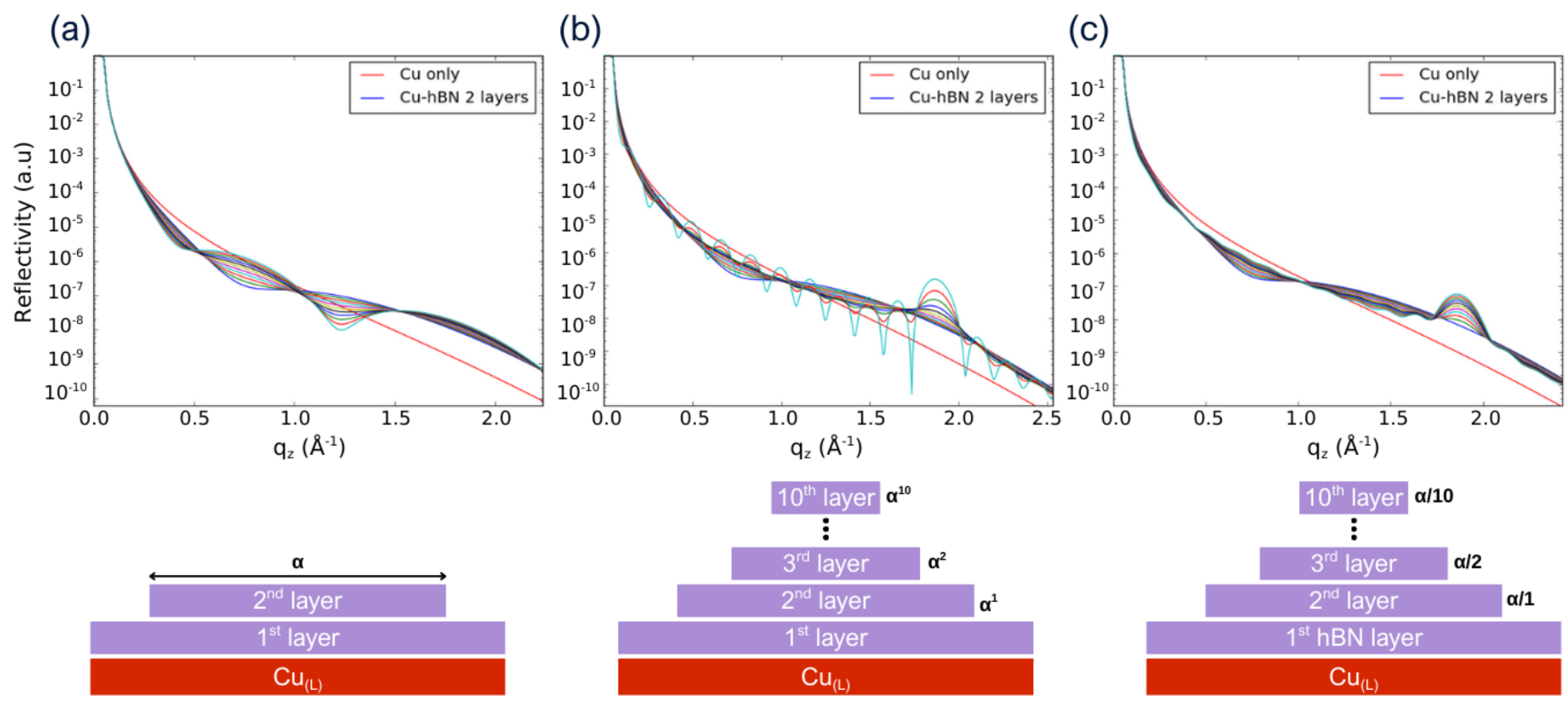

Figure S10: Simulated X-ray reflectivity (XRR) profiles of bare Liq-Cu and hBN multilayer structures on Liq-Cu: (a) A complete monolayer partially covered by an hBN terrace of lateral length α; (b) a monolayer topped with a pyramidal island composed of ten stacked hBN layers, where the lateral length of the n-th layer decreases exponentially as α^n; (c) same as (b), but with the lateral length decreasing linearly, such that the tenth layer's length is 10% of the second. The lateral length parameter α ranges from 0 to 1 in steps of 0.1. Schematic representations of models are shown below their respective curves.

Table S1: Growth parameters and XRR curve fitting parameters for best Lorentzian fits of out-of-plane peaks of hBN in Figure 3.

Label	Substrate	$T_{AB(max)}$ [°C]	t_{tot} [min]	Peak Center [$Å^{-1}$]	FWHM [$Å^{-1}$]	Interlayer Spacing [Å]	Out-of-plane Crystalline Domain Size [nm]	Approx. Number of Monolayers
Run I	Liq-Cu	85	50	-	-	-	-	1
Run II	Liq-Cu	85	50	1.783 ± 0.003	0.306 ± 0.019	3.523 ± 0.006	3.27 ± 0.20	6
Run III	Liq-Cu	94	29	1.786 ± 0.002	0.218 ± 0.015	3.518 ± 0.004	4.59 ± 0.31	8
Run IV	Liq-Cu	105	50	1.803 ± 0.003	0.138 ± 0.011	3.486 ± 0.006	7.26 ± 0.59	13
Run V	Liq-Cu	95	61	1.807 ± 0.001	0.117 ± 0.003	3.477 ± 0.001	8.58 ± 0.22	16
Run VI	SC-Cu	70	24	-	-	-	-	1
Run VII	SC-Cu	100	80	-	-	-	-	1
Run VIII	SC-Cu	105	113	-	-	-	-	1
Run IX	SC-Cu	120	138	-	-	-	-	1

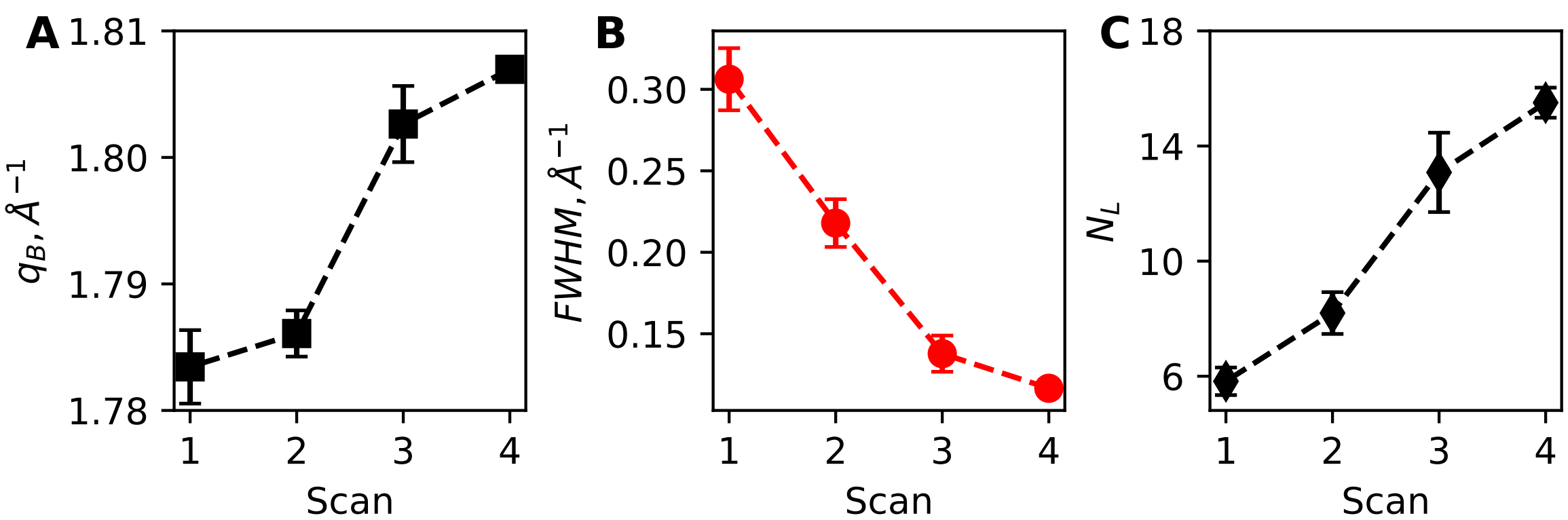

Figure S11: Parameters of the out-of-plane Bragg peak recorded on liquid Cu. A. Position q_B of the Bragg peak. B. FWHM of the Bragg peak C. Approximate number of layers $N_L = q_B/FWHM$ in the out-of-plane (q_z) direction.

GID.

To distinguish hBN from the Cu substrate in GID, one must consider their distinct scattering signatures. Bare Liq-Cu exhibits a broad scattering peak at scattering vector $q_{xy} \simeq 3Å^{-1}$ (Figure S12). This is typical for liquids as the absence of sharp peaks reflects the lack of long-range crystalline structure at the surface. SC-Cu exhibits crystal truncation rods (CTRs), formed by truncated 3D crystal scattering.[14,15] SC-Cu CTRs can be distinguished from hBN BRs at the same measurement position, as monolayer hBN is composed of lighter atoms (B and N) and thus generates significantly weaker scattering intensities, which decay with increasing q_z (the out-of-plane direction), wheras CTRs are more intense at higher q_z.[16] The formation of large single-crystalline Cu domains was confirmed by the detection of the intense and narrow Bragg

peaks upon slow re-solidification (see Figures S13 and S14 for more details).

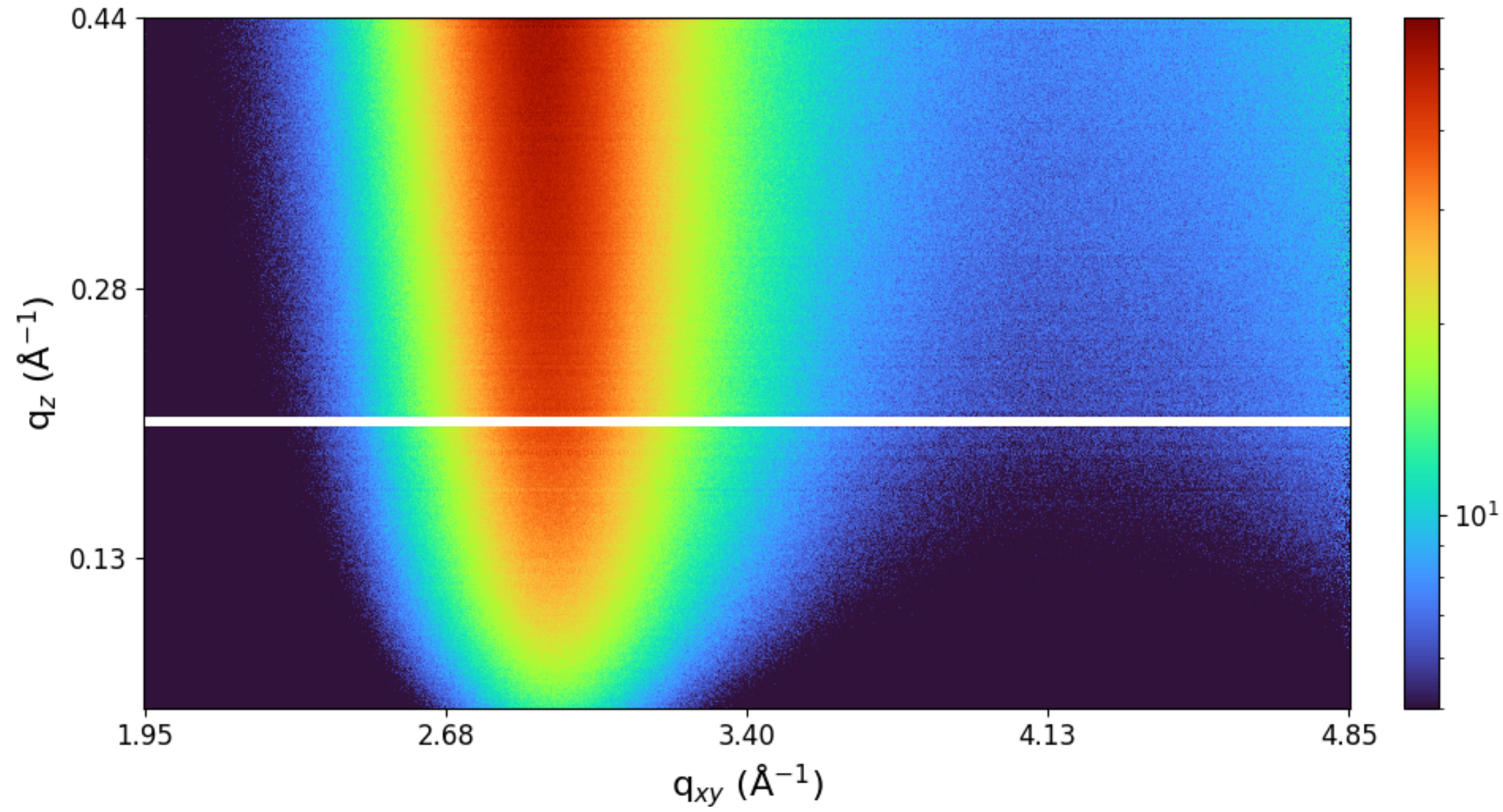

Figure S12: Reciprocal-space detector image of bare Liq-Cu obtained by stitching together a δ-scan (rotating the detector around the sample). The broad scattering peak is characteristic of a liquid due to the absence of long-range order. Measurements were taken at ID10-SURF beamline (ESRF) with an energy of 22.5 keV.

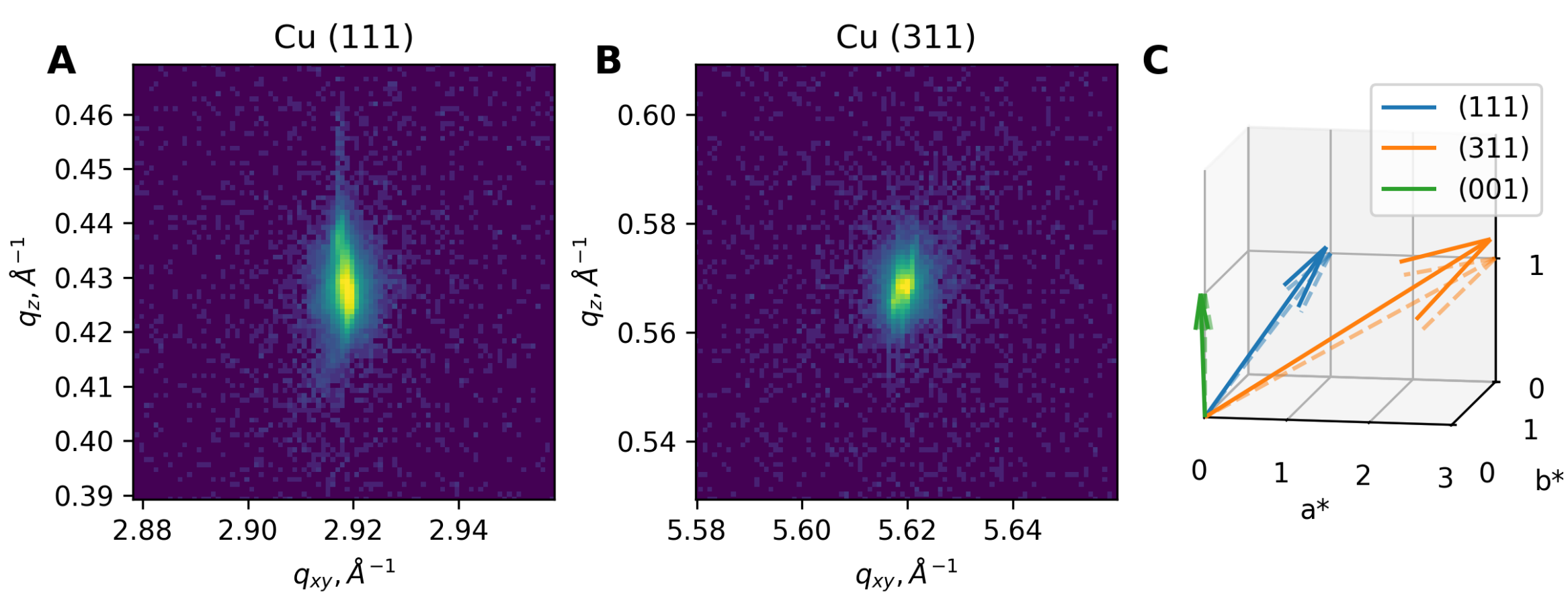

Figure S13: Orientation of the single-crystal Cu, after first crystallization. Sharp Bragg peaks were recorded for the solidified Cu, indicating formation of a single crystal. Diffraction was recorded in grazing-incidence geometry to maximize footprint on the sample. A. Position of (111) Bragg peak. B. Position of (311) Bragg peak. C. Schematic of crystal orientation, consistent with the recorded Bragg peaks originating from a single crystal. Note that the (001) peak is tilted ca. 3 degrees which allows for the (111) and (311) peaks to have slightly different q_z values. Lattice constant determined from recorded Bragg peaks $d = 3.68941 \pm 0.00017$ Å. This is in line with the literature value of $d_{Cu} = 3.6905$ Å at 1300 K.[17]

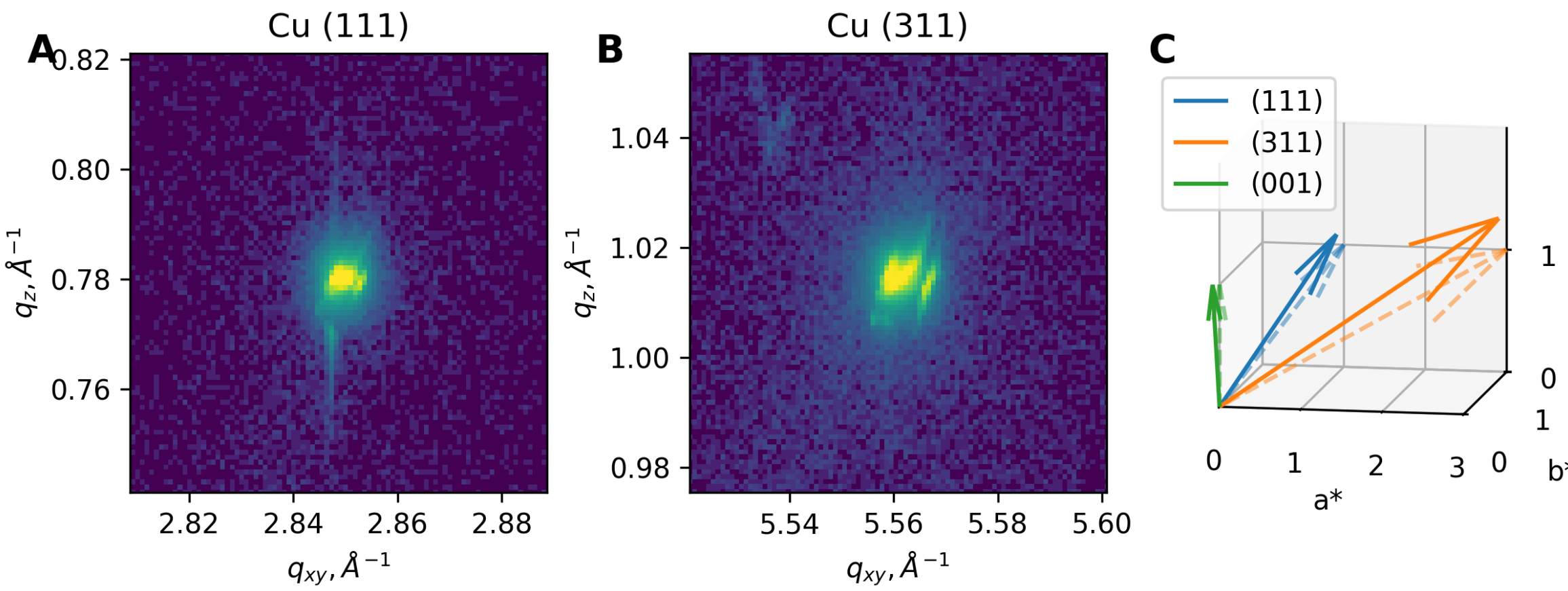

Figure S14: Orientation of the single crystal Cu, after second crystallization. Sharp Bragg peaks were recorded for the solidified Cu, indicating formation of a single crystal. Diffraction was recorded in grazing-incidence geometry to maximize footprint on the sample. A. Position of (111) Bragg peak. B. Position of (311) Bragg peak. C. Schematic of crystal orientation, consistent with the recorded Bragg peaks originating from a single crystal. Note that the (001) peak is tilted ca. 4.9 degrees which allows for the (111) and (311) peaks to have slightly different q_z values. Lattice constant determined from recorded Bragg peaks $d = 3.68537 \pm 0.00112$ Å. This is in line with the literature value of $d_{Cu} = 3.6858$ Å at 1250 K.[17]

BINoculars data analysis.

The individual scans comprising Bragg rods (BRs) can be projected onto three-dimensional reciprocal-space maps using the BINoculars binning algorithm.[18] In the resulting azimuthal q_x-q_y projections, each point represents the contribution of an individual crystallite to the measured BR signal. Figures S15 and S16 show the corresponding 3D reciprocal-space projections of [11] BRs on Liq-Cu and SC-Cu (Figure 4 (a, b)), respectively. To obtain the in-plane heatmaps shown in Figure 4 (g, h), the voxel intensities were summed along q_z. The effective voxel size is limited by the detector pixel size in real-space (0.055 µm) and the measurement step size of the azimuthal scan.

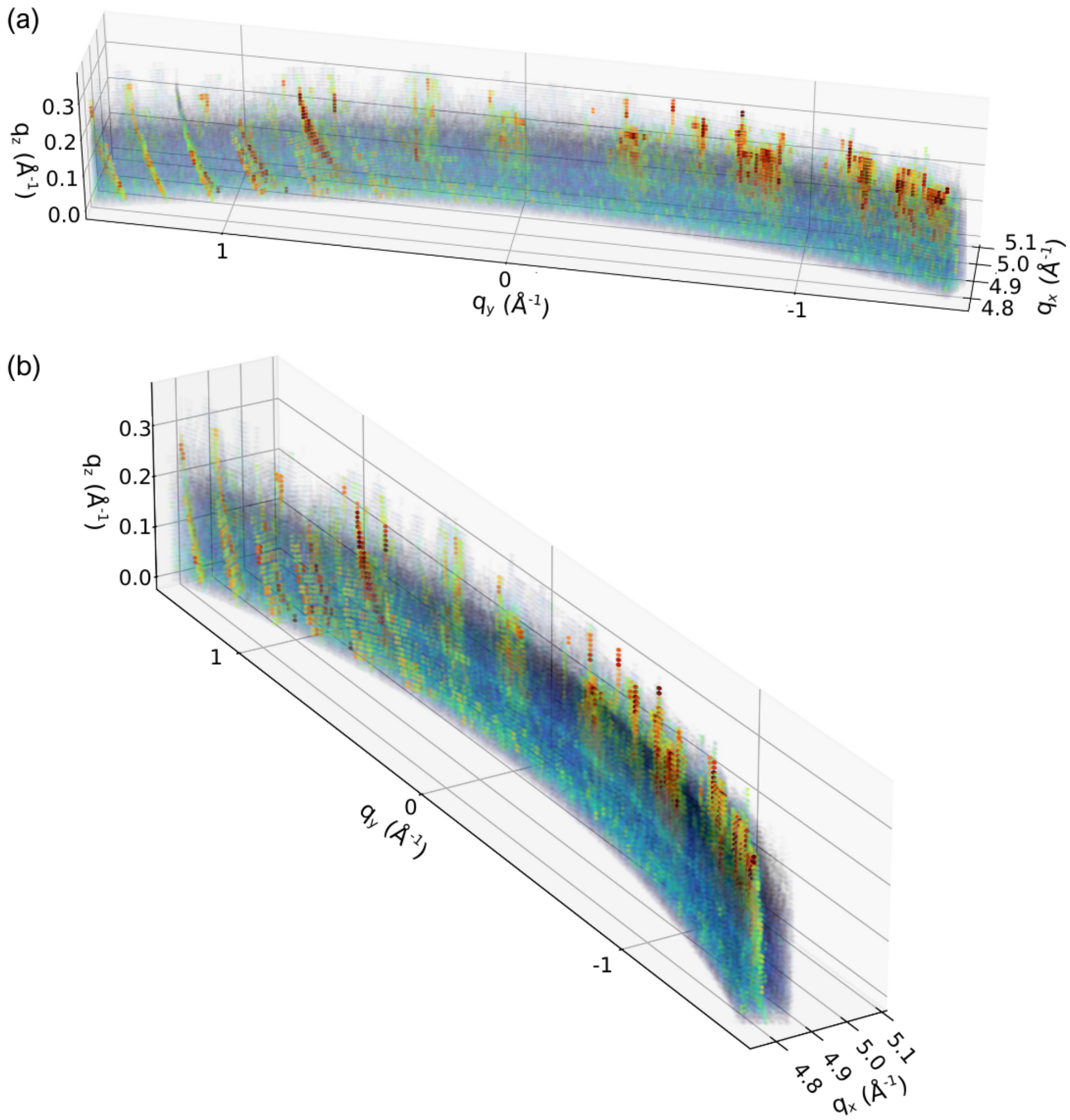

Figure S15: Exemplary in-plane (azimuthal) projection of the hBN/Liq-Cu [11] BRs comprising the summed image in Figure 4 (a) in 3D space. Intense regions (orange-red) correspond to individual diffracting crystallites and reflect their in-plane orientations.

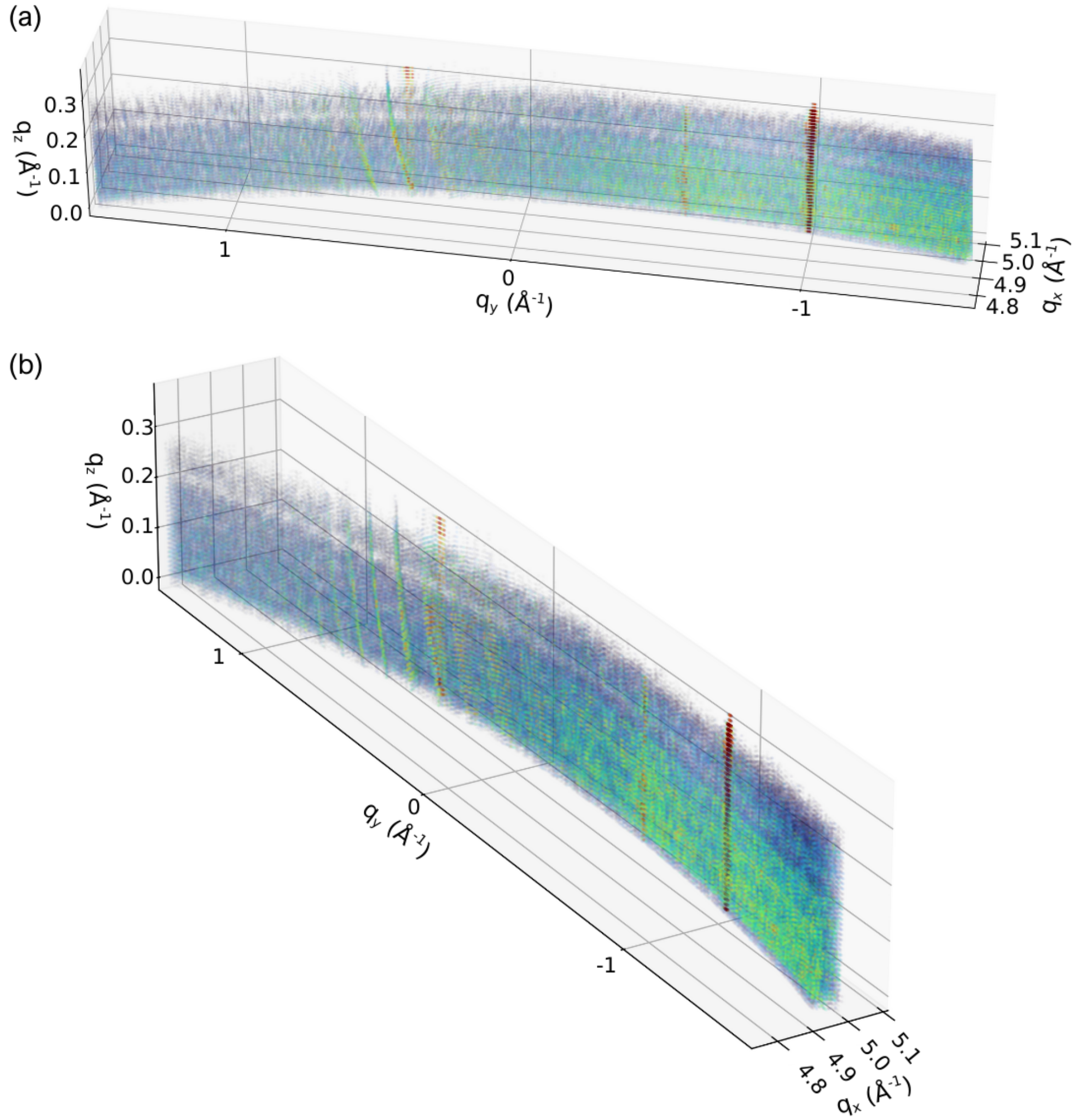

Figure S16: Exemplary in-plane (azimuthal) projection of the hBN/SC-Cu [11] BRs comprising the summed image in Figure 4 (b) in 3D space. The most intense (orange-red) values on the heat map correspond to individual diffracting grains and their in-plane positions.

FishingROD: GID data processing workflow.

2D maps in Figures S17 (a-f) and S18 (a-c) show hBN BRs which are composites of multiple images capturing scattering from individual grains while rotating a sample azimuthally (artificial powder averaging). q_{xy} and q_z correspond to in-plane and out-of-plane momentum transfers, respectively. To obtain the intrinsic FWHM of hBN BRs, the contribution of the measurement slit (σ_{slit} = 1 pixel) was deconvoluted from the fitted Gaussian width (σ_{fit}):

$$\sigma_{deconv} = \sqrt{\sigma_{fit}^2 - \sigma_{slit}^2} \tag{6}$$

From this FWHM, average grain sizes were estimated using the Scherrer approximation:[19]

$$\textbf{Domain size} = K\frac{2\pi}{FWHM} \tag{7}$$

where FWHM is in units of reciprocal space and K (=0.9) is a dimensionless shape factor. Measurement errors arising from the X-ray beam's footprint on the sample were estimated following the method described by Abbondanza et al.,[20] where the spread on the detector (x) is given as:

$$x = w \cdot \sin(2\theta) \tag{8}$$

where w ($\simeq$7.6 mm) is the beam's footprint on the sample and 2θ the diffraction angle at E = 22.5 keV (25.4° for hBN [11] and 14.6° for hBN [10]). This corresponds to an in-plane measurement uncertainty of ± 30 pixels for hBN [11] and ± 18 pixels for hBN [10], i.e. $\delta \mathrm{q_{xy}}^{[11]} = \pm 0.027\text{Å}^{-1}$ and $\delta \mathrm{q_{xy}}^{[10]} = \pm 0.017\text{Å}^{-1}$. These uncertainties were then combined with the fitting errors shown in Tables S2 and S3 to obtain the total errors reported in the main text. For further details on peak detection, background subtraction, and Gaussian fitting, see the openly available FishingROD software.[21] Overall distribution of the in-plane lattice parameters from all measurement runs with total error is shown in Figure S19.

We note that the [11] BR on Liq-Cu appears as an arc composed of multiple short, straight segments, likely originating from weak short-range correlations that manifest as elongated peaks from small crystallites randomly oriented under the beam. In contrast, the corresponding [11] BR on SC-Cu is narrow and straight, consistent with a uniform, 2D hBN layer with large grain size.[15] We ascribe these differences to the growth mode; on SC-Cu, the inherently static growth allows domains to anchor to the surface, resulting in a less misoriented texture with potentially larger domain sizes. Meanwhile on Liq-Cu, the high mobility and weak interaction of the hBN grains on the liquid surface, even within a densely packed layer, leads to a textured organization of smaller crystallites, with dense packing possibly also giving rise to jamming.

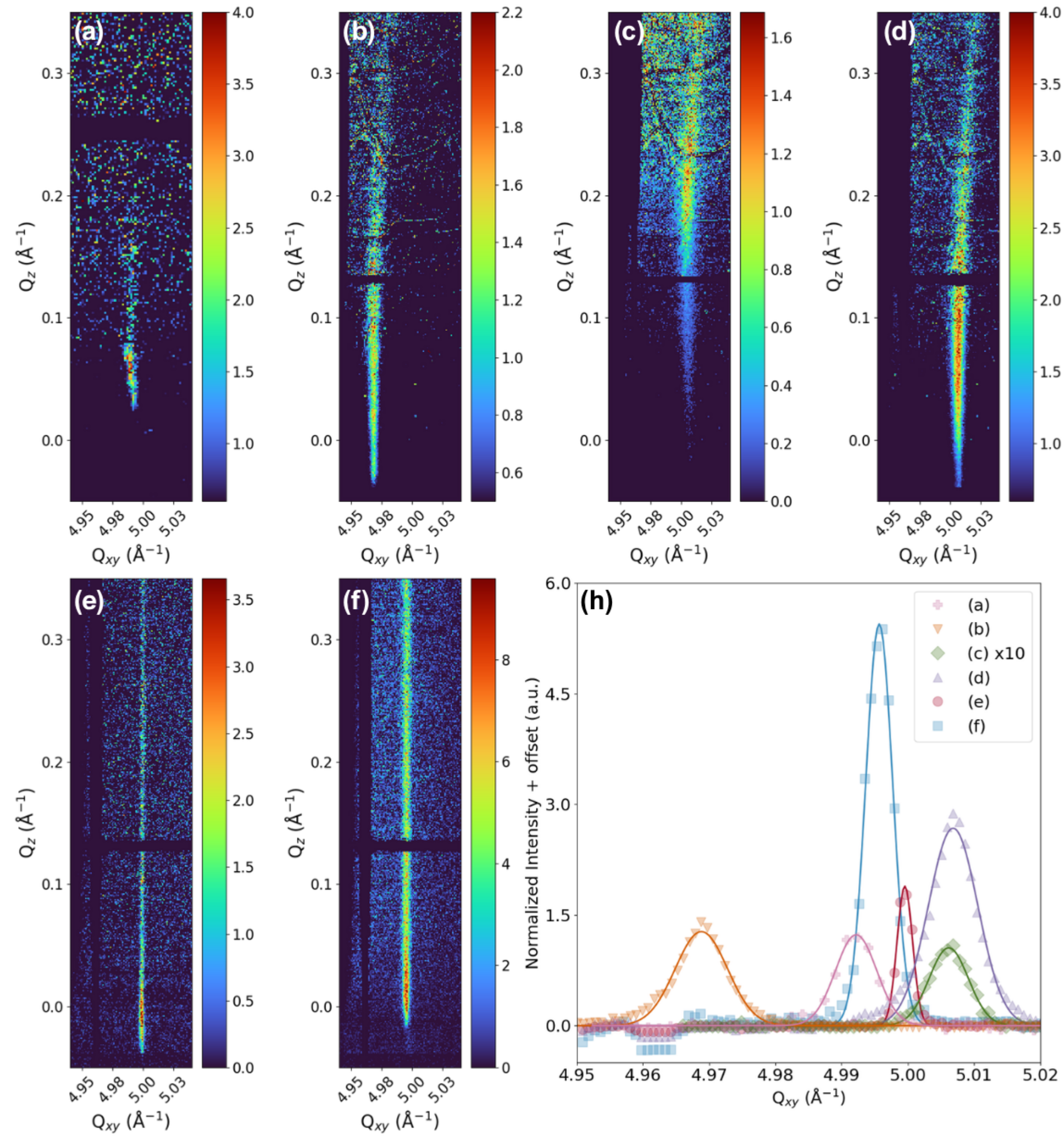

Figure S17: Reciprocal space detector images of hBN [11] 2D Bragg rods (BRs) for layers grown on Liq-Cu (a-d) and SC-Cu (e, f). Each image is a composite of multiple measurements taken while rotating the sample. (h) 1D signals of [11] BRs with Gaussian fits shown as solid lines. The signal from (c) has been scaled by ×10 for clarity. See Table S2 for more details.

Table S2: Best fit and derived data of [11] BRs shown in Figure S17.

Index	(a)	(b)	(c)	(d)	(e)	(f)
Substrate	Liq-Cu	Liq-Cu	Liq-Cu	Liq-Cu	SC-Cu	SC-Cu
# images averaged	57	326	250	165	10	8
Peak center [$Å^{-1}$]	4.992 ± 0.003	4.969 ± 0.004	5.006 ± 0.003	5.007 ± 0.004	5.000 ± 0.001	4.996 ± 0.002
a_1[Å]	2.517 ± 0.001	2.529 ± 0.002	2.511 ± 0.001	2.510 ± 0.002	2.514 ± 0.001	2.515 ± 0.001
FWHM [$Å^{-1}$]	0.0062	0.0091	0.0067	0.0086	0.0018	0.0043
Domain size [µm]	0.0913	0.0626	0.0847	0.0660	0.3130	0.1310

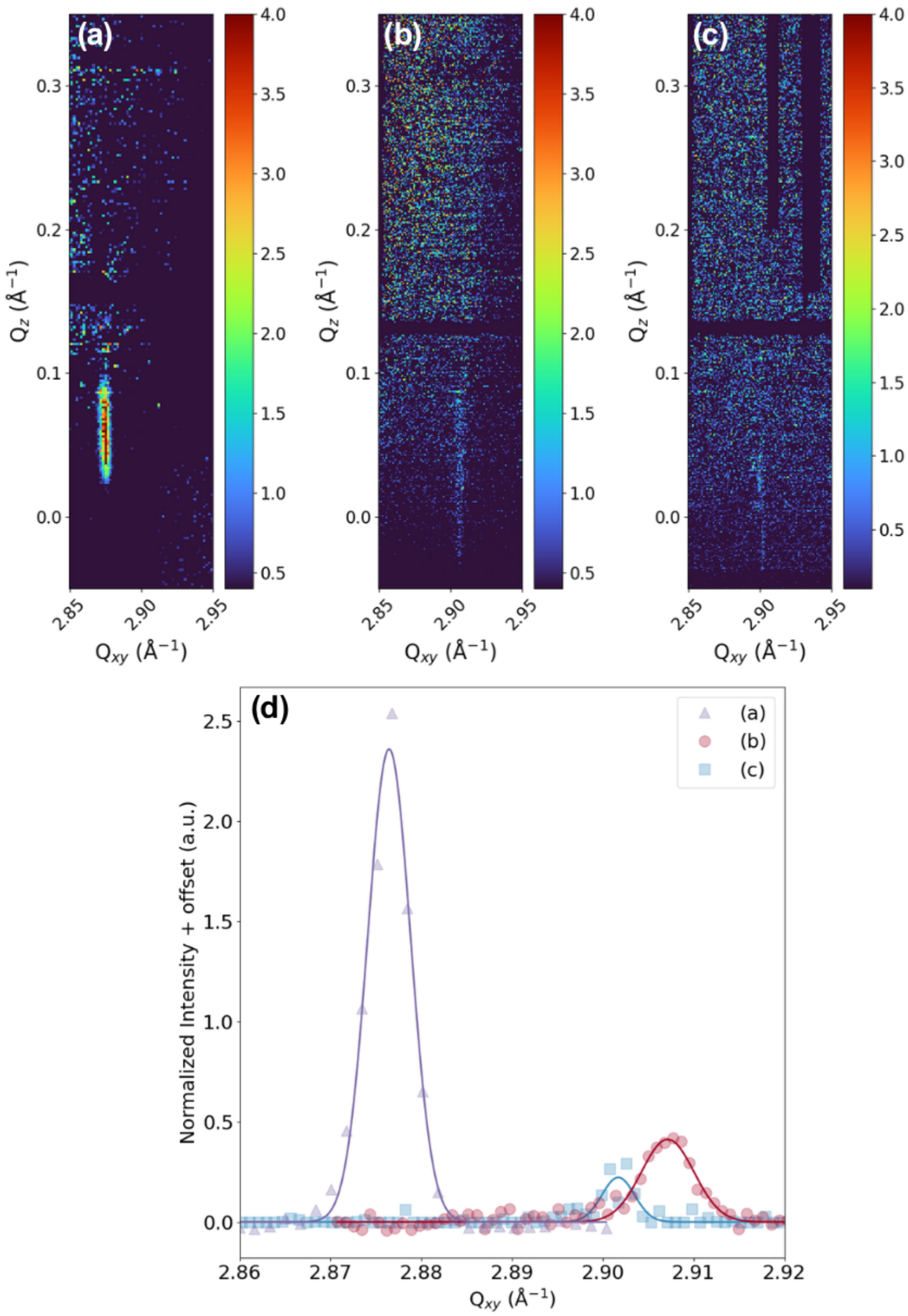

Figure S18: Reciprocal space detector images of hBN [10] 2D Bragg rods (BRs) for layers grown on Liq-Cu (a,b) and SC-Cu (c). Each image is a composite of multiple measurements taken while rotating the sample. (d) 1D signals of [10] BRs with Gaussian fits shown as solid lines. See Table S3 for more details.

Table S3: Best fit and derived data of [10] BRs shown in Figure S18.

Index	(a)	(b)	(c)
Substrate	Liq-Cu	Liq-Cu	SC-Cu
# images averaged	817	123	13
Peak center [$Å^{-1}$]	2.876 ± 0.002	2.907 ± 0.003	2.902 ± 0.001
a_1[Å]	2.522 ± 0.001	2.496 ± 0.002	2.500 ± 0.001
FWHM [$Å^{-1}$]	0.0039	0.0064	0.0034
Domain size [µm]	0.1444	0.0889	0.1659

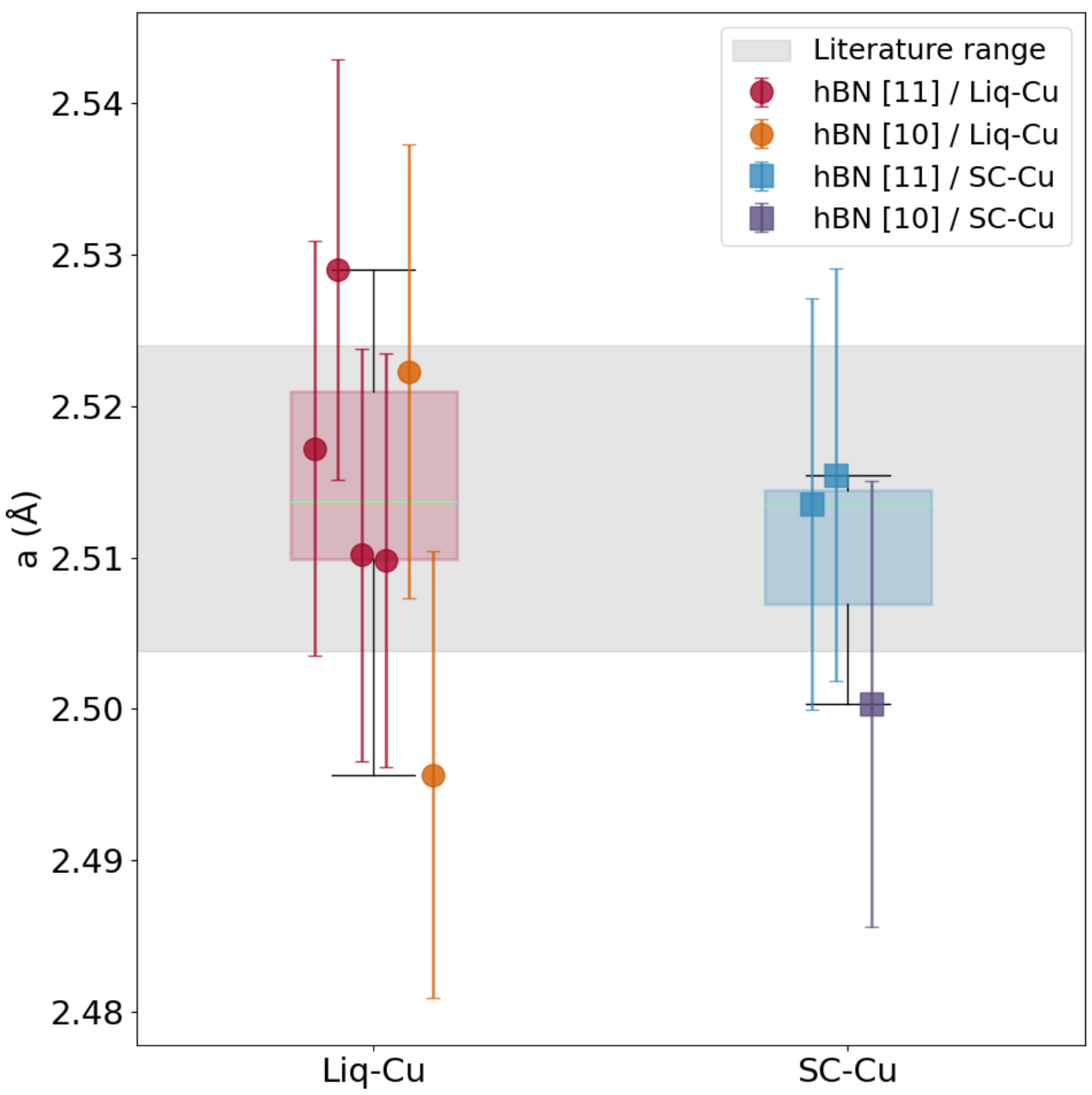

Figure S19: Distribution of the in-plane lattice parameter (a) for hBN obtained from the measured [11] and [10] BRs. Each semi-transparent box represents the overall spread of lattice parameters for Liq-Cu (red) and SC-Cu (blue) substrates, while individual data points correspond to distinct growth runs. Vertical bars indicate total uncertainties, accounting for both diffraction peak fitting and measurement errors. The shaded horizontal band (grey) denotes the lattice parameter range of hBN reported in the literature at room temperature (a = 2.504-2.524 Å).[22]

Experimental Electron Density Fitting.

Table S4: XRR curve fitting parameters for hBN grown on Liq-Cu and SC-Cu. Fixed parameters: density of Cu ρ_{Cu}, density of hBN ρ_{hBN}, thickness of hBN layer d_{hBN}, and the free ones: roughness of Cu σ_{Cu}, roughness of hBN σ_{hBN}, and thickness of the void slab d_{void}. The error values stem from averaging over several measurements.

	ρ_{Cu} [g·cm^{-3}]	σ_{Cu} [Å] (bare)	σ_{Cu} [Å] (covered)	ρ_{hBN} [g·cm^{-3}]	d_{hBN} [Å]	σ_{hBN} [Å]	d_{void} [Å]	d_{gap} [Å]
Liq-Cu	7.96	1.065 ± 0.032	1.043 ± 0.242	5.28	1.45	1.043 ± 0.242	1.72 ± 0.06	2.44 ± 0.06
SC-Cu	8.4	1.235 ± 0.078	1.212 ± 0.191	5.28	1.45	1.212 ± 0.191	1.66 ± 0.05	2.38 ± 0.05

MTP Fitting Details and Results.

The training set was adapted from a Gr-Cu dataset used in our previous work,[23] consisting of bulk and slab configurations for SC-Cu and Liq-Cu which in some cases are decorated with (strained) Gr that we replaced with hBN (see Table S5).

The trained MTP is very precise with root-mean-squared errors of 1.8 meV/atom (2.2 meV/atom) in energies and 51 meV/Å^{-1} (68 meV/Å^{-1}) in the forces for the training (and test) set (see Figure S20).

Table S5: List of configurations used for training the MLIP describing the hBN/Cu interface. N_{train} and N_{test} represent the number of configurations contained in the training and test set, respectively. The data set is analogous to one for an MLIP to describe the Gr/Cu interface,[23] but omits one large structure composed of a Liq-Cu slab and a Gr flake, which does not significantly improve the accuracy of MTP.

Description	Formula	N_{train}	N_{test}
fcc Cu (with different lattice constants)	Cu_4	4	1
fcc Cu (222 supercell, rattled)	Cu_{32}	4	1
Cu(111) (444 supercell, rattled)	Cu_{64}	4	1
hBN on Cu(111) (varying hBN height)	$B_{16}N_{16}Cu_{64}$	16	4
hBN liquid Cu slab (varying hBN height)	$B_{16}N_{16}Cu_{64}$	16	4
hBN (with different lattice constants)	BN	4	1
hBN (441 supercell, rattled)	$B_{16}N_{16}$	4	1
Liquid Cu	Cu_{108}	4	1
Liquid Cu slab	Cu_{64}	4	1

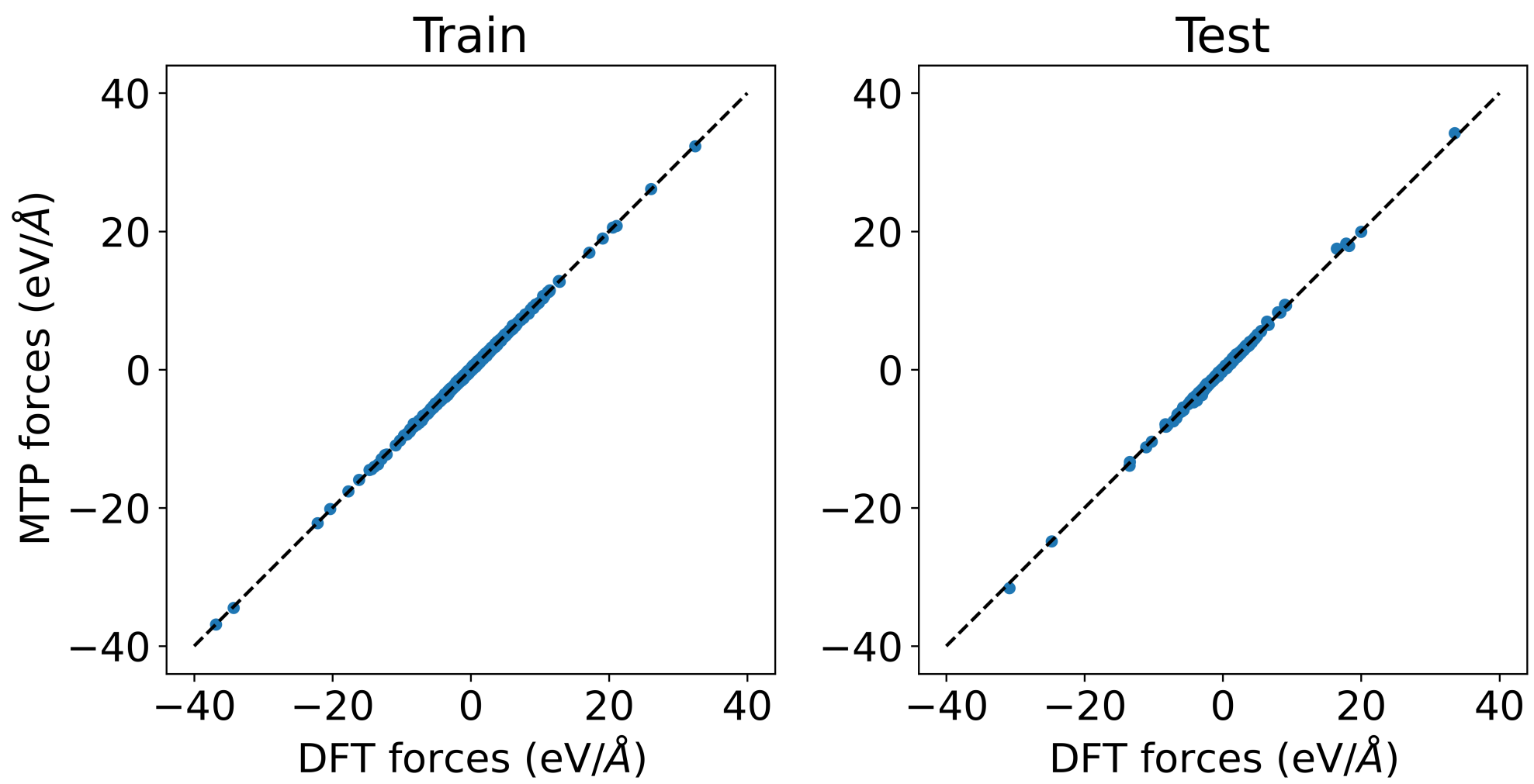

Figure S20: Correlation plot for forces predicted by MTP and DFT on both training (left) and test (right) datasets.

Table S6: Lattice parameters of hBN and Cu from experiments, DFT, and MTP.

Lattice parameter (Å)	Experiment	PBE+MBD-nl	MTP
a_{hBN}	2.504 (293.15K)[24]	2.514	2.515
a_{Cu}	3.597[25]	3.600	3.594

Details of Simulation Models.

In the case of Liq-Cu, a zero-strain model was realized by simply adjusting the simulation cells' surface-parallel dimensions according to the temperature-dependent lattice parameters of either hBN or Gr. The liquid Cu can freely align to these cell dimensions, resulting in "small" models with formula units $B_{168}N_{168}Cu_{1344}$ and $C_{364}Cu_{1344}$. In the case of SC-Cu, which we represented via Cu(111), surface-parallel dimensions were adjusted according to the temperature-dependent lattice parameters of fcc-Cu and very large simulation cells were prepared that can accommodate almost commensurate 2D-sheets with minimal strain of $\lesssim |\pm 0.4\%|$ (compare Table S9 and S8).

To exclude a possible influence of any residual stress in the SC-Cu models, two sets of simulation cells were build per system corresponding to $B_{1458}N_{1458}Cu_{10816}$ and $B_{1568}N_{1568}Cu_{11664}$

as well as $C_{1156}Cu_{4096}$ and $C_{1296}Cu_{4624}$. For both hBN and Gr, the two simulation cells yielded minimal deviation and a negligible temperature dependence (compared to simulation cells with larger stress[23]) which attested a marginal influence of the residual stress (see Table S9 and S8).

The DFT models used for interfacial electronic-structure analysis consist of a $1\times\sqrt{3}$ Cu(111) orthogonal supercell with 6 Cu layers and either hBN or graphene adsorbed on one side of the slab (corresponding to stoichiometries $B_2N_2Cu_{12}$ and C_4Cu_{12}, respectively). The Cu bulk lattice constant was optimized in a primitive fcc unit cell using a Burch-Murnaghan equation-of-state fit, and all slab geometries were subsequently relaxed using the conjugate gradient algorithm until forces converged below 50 meV/Å. These minimal slab models are intentionally idealized and, accordingly, the resulting insights should be interpreted qualitatively, as they are intended to enable a systematic comparative assessment of interfacial trends rather than a quantitatively predictive description.

hBN/Gr geometry analysis during MD.

Table S7: Mean values and standard deviations (std) of out-of-plane deformation distributions (compare Figure 6 in the main text).

System	Atom	Mean (Å)	Std (Å)
Freestanding hBN (1370 K)	B	10^{-5}	0.0955
hBN/Liq-Cu (1370 K)	B	-0.0152	0.1035
Freestanding hBN (1300 K)	B	6×10^{-5}	0.0929
hBN/SC-Cu (1300 K)	B	-0.0157	0.1008
Freestanding hBN (1370 K)	N	-2×10^{-5}	0.1434
hBN/Liq-Cu (1370 K)	N	0.0148	0.1449
Freestanding hBN (1300 K)	N	-7×10^{-5}	0.1390
hBN/SC-Cu (1300 K)	N	0.0153	0.1398
Freestanding Gr (1370 K)	C	3×10^{-6}	0.1000
Gr/Liq-Cu (1370 K)	C	1×10^{-4}	0.1111
Freestanding Gr (1300 K)	C	-1×10^{-6}	0.0971
Gr/SC-Cu (1300 K)	C	-5×10^{-5}	0.1053

Table S8: Separation distance (gap) between hBN and Cu surfaces processed from MD simulations of model systems at different temperatures, phases, and, strains. In the main text, we reported gap values of the model with the least strain.

T (K)	Solid/Liquid	hBN strain (%)	Formula	Gap (Å)
1370	Liquid	0	$B_{168}N_{168}Cu_{1344}$	2.350
1300	Solid	-0.12	$B_{1458}N_{1458}Cu_{10816}$	2.301
1300	Solid	+0.02	$B_{1568}N_{1568}Cu_{11664}$	2.292
1200	Solid	-0.38	$B_{1458}N_{1458}Cu_{10816}$	2.303
1200	Solid	-0.24	$B_{1568}N_{1568}Cu_{11664}$	2.302

Table S9: Separation distance (gap) between Gr and Cu surfaces processed from MD simulations of model systems at different temperatures, phases, and strains. In the main text, we reported gap values of the model with the least strain.

T (K)	Solid/Liquid	Gr strain (%)	Formula	Gap (Å)
1370	Liquid	0	$C_{364}Cu_{1344}$	2.111
1300	Solid	-0.2	$C_{1156}Cu_{4096}$	2.117
1300	Solid	+0.14	$C_{1296}Cu_{4624}$	2.110
1200	Solid	-0.42	$C_{1156}Cu_{4096}$	2.139
1200	Solid	-0.07	$C_{1296}Cu_{4624}$	2.126

Table S10: Difference between averaged heights of B $\langle h_B \rangle$ and N $\langle h_B \rangle$ with and without Cu surfaces.

	hBN (1300 K)	hBN (1370 K)	hBN/SC-Cu (1300 K)	hBN/Liq-Cu (1370 K)
$\langle h_B \rangle - \langle h_N \rangle$ (Å)	6×10^{-6}	7×10^{-6}	-0.0152	-0.0147

Table S11: Hirshfeld charges ($|e|$) for hBN, Gr, Cu(111), and their interfaces. C1 and C2 represent two types of carbon with different environments in Gr/SC-Cu.

	C1	C2	B	N	Cu (surface)	Cu (2nd layer)
Cu(111)	–	–	–	–	-0.006	$+0.006$
Gr (free-standing)	$< 10^{-4}$	$< 10^{-4}$	–	–	–	–
Gr/SC-Cu	-0.008	-0.006	–	–	$+0.008$	$+0.006$
hBN (free-standing)	–	–	$+0.2$	-0.2	–	–
hBN/SC-Cu	–	–	$+0.18$	-0.2	$+0.011$	$+0.006$

References

(1) Babenko, V.; Lane, G.; Koos, A. A.; Murdock, A. T.; So, K.; Britton, J.; Meysami, S. S.; Moffat, J.; Grobert, N. Time dependent decomposition of ammonia borane for the controlled production of 2D hexagonal boron nitride. *Scientific Reports* **2017**, *7*, 14297.

(2) Saedi, M.; de Voogd, J. M.; Sjardin, A.; Manikas, A.; Galiotis, C.; Jankowski, M.; Renaud, G.; La Porta, F.; Konovalov, O.; van Baarle, G. J. C.; Groot, I. M. N. Development of a reactor for the in situ monitoring of 2D materials growth on liquid metal catalysts, using synchrotron x-ray scattering, Raman spectroscopy, and optical microscopy. *Review of Scientific Instruments* **2020**, *91*, 013907.

(3) Stehle, Y.; Meyer III, H. M.; Unocic, R. R.; Kidder, M.; Polizos, G.; Datskos, P. G.; Jackson, R.; Smirnov, S. N.; Vlassiouk, I. V. Synthesis of Hexagonal Boron Nitride Monolayer: Control of Nucleation and Crystal Morphology. *Chemistry of Materials* **2015**, *27*, 8041–8047.

(4) Wu, C.; Soomro, A. M.; Sun, F.; Wang, H.; Huang, Y.; Wu, J.; Liu, C.; Yang, X.; Gao, N.; Chen, X.; Kang, J.; Cai, D. Large-roll growth of 25-inch hexagonal BN monolayer film for self-release buffer layer of free-standing GaN wafer. *Scientific Reports* **2016**, *6*, 34766.

(5) Park, J.-H.; Lu, A.-Y.; Tavakoli, M. M.; Kim, N. Y.; Chiu, M.-H.; Liu, H.; Zhang, T.; Wang, Z.; Wang, J.; Martins, L. G. P.; Luo, Z.; Chi, M.; Miao, J.; Kong, J. Revealing Variable Dependences in Hexagonal Boron Nitride Synthesis via Machine Learning. *Nano Letters* **2023**, *23*, 4741–4748, PMID: 37196055.

(6) Rein, V.; Gao, H.; Heenen, H. H.; Sghaier, W.; Manikas, A. C.; Tsakonas, C.; Saedi, M.; Margraf, J. T.; Galiotis, C.; Renaud, G.; Konovalov, O. V.; Groot, I. M. N.; Reuter, K.; Jankowski, M. Operando Characterization and Molecular Simulations Reveal the Growth Kinetics of Graphene on Liquid Copper During Chemical Vapor Deposition. *ACS Nano* **2024**, *18*, 12503–12511, PMID: 38688475.

(7) Zhang, D.; Yi, P.; Lai, X.; Peng, L.; Li, H. Active machine learning model for the dynamic simulation and growth mechanisms of carbon on metal surface. *Nature Communications* **2024**, *15*, 344.

(8) Trehan, R.; Lifshitz, Y.; Rabalais, J. W. Auger and x-ray electron spectroscopy studies of hBN, cBN, and N+2 ion irradiation of boron and boron nitride. *Journal of Vacuum Science Technology A* **1990**, *8*, 4026–4032.

(9) Song, L.; Ci, L.; Lu, H.; Sorokin, P. B.; Jin, C.; Ni, J.; Kvashnin, A. G.; Kvashnin, D. G.; Lou, J.; Yakobson, B. I.; Ajayan, P. M. Large Scale Growth and Characterization of Atomic Hexagonal Boron Nitride Layers. *Nano Letters* **2010**, *10*, 3209–3215, PMID: 20698639.

(10) Musket, R.; McLean, W.; Colmenares, C.; Makowiecki, D.; Siekhaus, W. Preparation of atomically clean surfaces of selected elements: A review. *Applications of Surface Science* **1982**, *10*, 143–207.

(11) Febvre, P.; Taillet, R.; Villain, L. *Dictionnaire de physique*; De Boeck superieur: Louvain-La-Neuve, Belgium, 2013; p 575.

(12) Kondrat'ev, Y.; Butlak, A.; Kazakov, I.; Timoshkin, A. Sublimation and thermal decomposition of ammonia borane: Competitive processes controlled by pressure. *Thermochimica Acta* **2015**, *622*, 64–71, Applications of Thermal Analysis techniques for sublimation and vaporization studies.

(13) Amar, E.; Queirós, T.; Nicoara, N.; Nemala, S. S.; Garzón, D. A.; Peters, J. C.; Nieder, J. B.; Alpuim, P.; Tavares, C. J.; Sadewasser, S. Effect of ammonia borane thermal decomposition under different Ar fluxes on large-area boron nitride films for quantum photonic applications. *Mater. Adv.* **2026**, *7*, 2441–2453.

(14) Robinson, I. K.; Tweet, D. J. Surface X-ray diffraction. *Rep. Prog. Phys.* **1992**, *55*, 599–651.

(15) Chen, H. Surface/interface X-ray diffraction. *Materials Chemistry and Physics* **1996**, *43*, 116–125.

(16) Willmott, P. *An Introduction to Synchrotron Radiation*; John Wiley Sons, Ltd, 2019; Chapter 2, pp 19–49.

(17) Arblaster, J. W. *Selected values of the crystallographic properties of elements*; A S M International: Materials Park, 2018.

(18) Roobol, S.; Onderwaater, W.; Drnec, J.; Felici, R.; Frenken, J. *BINoculars*: data reduction and analysis software for two-dimensional detectors in surface X-ray diffraction. *Journal of Applied Crystallography* **2015**, *48*, 1324–1329.

(19) Scherrer, P. Bestimmung der Größe und der inneren Struktur von Kolloidteilchen mittels Röntgenstrahlen. *Nachrichten von der Gesellschaft der Wissenschaften zu Göttingen, Mathematisch-Physikalische Klasse* **1918**, *1918*, 98–100.

(20) Abbondanza, G.; Larsson, A.; Carlá, F.; Lundgren, E.; Harlow, G. S. Quantitative powder diffraction using a (2+3) surface diffractometer and an area detector. *Journal of Applied Crystallography* **2021**, *54*, 1140–1152.

(21) Ghanadan, N.; Sarrazin, T. FishingROD [Software]. `https://github.com/Nikoo-Ghn/FishingROD`.

(22) Paszkowicz, W.; Pelka, J. B.; Knapp, M.; Szyszko, T.; Podsiadlo, S. Lattice parameters and anisotropic thermal expansion of hexagonal boron nitride in the 10–297.5 K temperature range. *Applied Physics A* **2002**, *75*, 431–435.

(23) Gao, H.; Belova, V.; La Porta, F.; Cingolani, J. S.; Andersen, M.; Saedi, M.; Konovalov, O. V.; Jankowski, M.; Heenen, H. H.; Groot, I. M. N.; Renaud, G.; Reuter, K. Graphene at Liquid Copper Catalysts: Atomic-Scale Agreement of Experimental and First-Principles Adsorption Height. *Advanced Science* **2022**, *9*, 2204684.

(24) Lynch, R. W.; Drickamer, H. G. Effect of High Pressure on the Lattice Parameters of Diamond, Graphite, and Hexagonal Boron Nitride. *44*, 181–184.

(25) Davey, W. P. Precision Measurements of the Lattice Constants of Twelve Common Metals. *25*, 753–761.
